# Supplementary material for: Impact of the use of GeneXpert on TB diagnosis and anti-TB treatment outcome at health facilities in Addis Ababa, Ethiopia in the post-millennium development years
Source: PLoS One. 2023 Aug 25;18(8):e0289917. doi: 10.1371/journal.pone.0289917 (PMC10456184; doi:10.1371/journal.pone.0289917)
Supplement: Attachment — Submitted filename: PONE-D-21-40508_R3_reviewer (2).pdf [file pone.0289917.s005.pdf]

PLOS ONE

Impact of GeneXpert on TB Diagnosis and Anti-TB Treatment Outcome at Health Facilities in Addis Ababa, Ethiopia: In the Post MDG Years

--Manuscript Draft--

**Manuscript Number:**	PONE-D-21-40508R3
**Article Type:**	Research Article
**Full Title:**	Impact of GeneXpert on TB Diagnosis and Anti-TB Treatment Outcome at Health Facilities in Addis Ababa, Ethiopia: In the Post MDG Years
**Short Title:**	Impact of GeneXpert on TB Diagnosis and Anti-TB Treatment Outcome in Addis Ababa, Ethiopia
**Corresponding Author:**	Desalegn Addise Getahun<br>Ethiopian Public Health Institute<br>Addis Ababa, ETHIOPIA
**Keywords:**	Tuberculosis, Treatment outcomes, GeneXpert, MDG, SDG, Addis Ababa, Ethiopia
**Abstract:**	Background: GeneXpert is an effective and rapid molecular system used for tuberculosis (TB) diagnosis. It is expected to improve the detection rate and treatment outcome to meet the sustainable development goal (SDG) and End TB strategy target set for 2030. This study aimed to evaluate the impact of GeneXpert on diagnosis and anti-TB treatment outcomes in the post-millennium development goals (MDGs) in the capital city of Ethiopia. Hence, the global priority indicator based on the End TB Strategy for TB treatment success rate was met early in 2018 at Addis Ababa, Ethiopia, which is anticipated to be met by 2025.<br>Method: A retrospective health facilities-based study was conducted in Addis Ababa, Ethiopia. Records of all TB cases diagnosed and treated in selected health facilities from January 2015 to December 2018 were reviewed and included in the study. Data analysis of descriptive and inferential statistics was conducted using SPSS version 20.<br>Result: The reviewed records have showed that a total of 45,158 presumptive pulmonary TB (PTB) cases had accessed TB diagnosis services. Of which, 28.9% (13072/45158) were tested by AFB microscopy and 71.1% (32086/45158) were tested by GeneXpert. In the study period, the coverage of Xpert MTB/RIF testing was increased to 94.9% in 2018 compared to 1.6% in 2015. The number of presumptive PTB cases, who tested with GeneXpert system compared to smear microscopy showed a significant increase. The odds of positivity were detected in males compared to females. The odds of detecting TB cases was much higher among study participants aged 15-44 years compared to younger than 15 years. Treatment success rate showed a relative improvement each year between 2015 and 2018 with a mean of 92.6%. Reduced odds of treatment successes were observed in age categories older than 35 years, and in TB/HIV co-infected patients. Increased odds of treatment successes were reported in the years between 2016 and 2018 compared to 2015.<br>Conclusion: Scaling up the Xpert MTB/RIF assay as a point of care test for presumptive TB cases in the resource-limited settings would have a significant impact to meet the SDG and End TB strategy both in the improvement of TB detection rate and positive treatment success rate.
**Order of Authors:**	Desalegn Addise Getahun
	Laura E. Layland
	Achim Hoerauf
	Biniam Wondale
**Response to Reviewers:**	1.Can the author explain the discrepancy between the date of the implementation of GeneXpert (2017) and the data which include 2015 and 2016<br><br>Response: Thank you for the comments.<br>We, the authors, would like to make clear the discrepancy related to the implementation of GeneXpert in Ethiopia;<br>•It is true that the GeneXpert instruments were introduced in Ethiopia and in use since 2012 (Alemu et al., 2019), to diagnose only selected presumptive TB cases

Powered by Editorial Manager® and ProduXion Manager® from Aries Systems Corporation

(Presumptive DR-TB, HIV +ve, Children; and EPTB), while the remaining cases with low-risk to DR-TB, HIV-VE, and adult were diagnosed by AFB microscopy based on the algorithm (FMoH Ethiopia, 2014). But, since 2017, GeneXpert system is in use to diagnose all presumptive TB cases presented with signs and symptoms of TB at any given health facility in Addis Ababa.
•It is because of selective TB diagnosis made by GeneXpert instruments were available in the years 2015 and 2016.

2.Can the author confirm the methodology - here a flow diagram may be useful - outlining the sensitivity and specificity of microscopy as well as the exact staining and review process used
Response: Thank you very much for the comments.
The sensitivity and specificity were not assessed since, in this investigation, we collected retrospective data from health facilities in Addis Ababa.
Both staining techniques (Ziel-Neelsen staining (ZN) or fluorescent auramine staining (LED FM)) were in use in Addis Ababa but the health facilities do not keep track of which staining techniques were employed; instead, the record indicates that the diagnosis was performed using sputum smear microscopy. Due to the lack of clarity in the registration books, we are unable to determine the exact staining techniques.

3.This manuscript still requires extensive English language editing
Response: Thank you for the comments and suggestions, and we made corrections per the comments.

4.Reviewer #3: Data in Table 1 shows an increase in TB testing (Total), I would like to know the reasons for the increase uptake from 4 635 in year 2015 to 17 572 in Year 2018. Secondly, the method for diagnosing using smear, was it a single smear per participant? if so why do we see an increase in uptake as GeneXpert testing is introduced.
Response: Thank you for the comments.

•It is true that since the GeneXpert instrument has been used for diagnosis of presumptive TB cases as a first-line test in the chosen health facilities in Addis Ababa, the number of presumptive TB cases diagnosed for TB has increased. The instrument's improved sensitivity and specificity, which increase the trust of clinicians working in the OPD, ease of use, the lack of a requirement for two sputum samples, and the suitability of the reagent/cartridge may all be contributing factors to the increased TB test.
•Based on the recommendation of the guideline for clinical and programmatic management of TB and TB/HIV in Ethiopia, two sputum samples (Spot-Spot) are required for AFB Microscopy, and one sputum smear positive result confirms TB diagnosis. In this study, we collected the final AFB microscopy result of the patients from the laboratory registration book.

5.Reviewer #4: - There are serious issues with style and grammar that affect comprehension and require editing

- There is inconsistency between information given in background and results: the background states that GXP was introduced in 2017, yet GXP data is presented from 2015 onwards

- The background lacks information on microscopy and GXP in terms of performance data/limit of detection

- The method section is flawed; there is no information on MTB testing algorithms in the laboratories; no information on which AFB microscopy method was used; no information on treatment regimens for MDR TB; extracted data and analysis thereof is not clearly stated

- The results section doesn't mention proportion of patients on DS or MDR treatment; patients with extra-pulmonary TB are included yet the methods section only mentions sputum samples-- how were these diagnosed and from which samples?; the impact of the GXP introduction on treatment outcomes is not mentioned in results and should be elaborated on; relevance of adding data for various 'sub-cities' not clear unless of local

	interest<br><br>Response:Thank you for the comments.<br><br>•Thank you for the comments and we made corrections per the comments.<br>•It is true that the GeneXpert instruments were introduced in Ethiopia and in use since 2012 (Alemu et al., 2019), to diagnose only selected presumptive TB cases (Presumptive DR-TB, HIV +ve, Children; and EPTB), while the remaining cases with low-risk to DR-TB, HIV-VE, and Adult are diagnosed by AFB microscopy based on the algorithm (FMoH Ethiopia, 2014). But, since 2017, GeneXpert system is in use to diagnose all presumptive TB cases presented with signs and symptoms of TB at any given health facility in Addis Ababa. It is because of selective TB diagnoses made by GeneXpert instruments were available in the years 2015 and 2016.<br>•The performance data is included on page 3 in the last paragraph (we added a sentence which describes the performance of AFB smear microscopy)<br>•Both staining techniques (Ziel-Neelsen staining (ZN) or fluorescent auramine staining (LED FM)) are in use in Addis Ababa, but the health facilities do not keep track of which staining techniques were employed; instead, the record indicates that the diagnosis was performed using sputum smear microscopy. Due to the lack of clarity in the laboratory registration books, we are unable to determine the exact staining techniques.<br>•65 MDR patients who were moved to the MDR Treatment Initiating Center (TIC) for second-line therapy and are not included in this study, as noted under Table 4 on page 10. Therefore, we were unable to obtain their treatment records. As a result, MDR patients are excluded from the treatment outcomes analysis.<br>•Clinical diagnosis was the main method of diagnosis for extra-pulmonary TB patients. Based on the recorded data only 10 (0.3%) of the 3694 extra-pulmonary TB patients had laboratory diagnostic information, The data record simply states that the diagnosis was made using either smear microscopy or GeneXpert, the health facilities did not keep track of the sample types utilized. That is why we don't give due attention on the diagnosis of EPTB and we are unable to pinpoint the precise sample type utilized.<br>•The result section on page 10 and lines 10th and 11th discuss the impact of the GXP introduction on treatment results.<br>•We appreciate your concern and your suggestion, adding data for various 'sub-cities' will be pertinent to local interest.
**Additional Information:**	
**Question**	**Response**
**Financial Disclosure**<br><br>Enter a financial disclosure statement that describes the sources of funding for the work included in this submission. Review the submission guidelines for detailed requirements. View published research articles from *PLOS ONE* for specific examples.<br><br>This statement is required for submission and **will appear in the published article** if the submission is accepted. Please make sure it is accurate.	The first author, Desalegn Addise, was funded by the Uni Bonn for the Master's program that helped enable him to conduct this study and Achim Hoerauf is also funded by the Deutsche Forschungsgemeinschaft (DFG, German Research Foundation) under Germany's Excellence Strategy – EXC2151 – 390873048. The funders had no role in study design, data collection and analysis, decision to publish, or preparation of the manuscript.

**Unfunded studies**<br>Enter: *The author(s) received no specific funding for this work.*<br><br>**Funded studies**<br>Enter a statement with the following details:<br>• Initials of the authors who received each award<br>• Grant numbers awarded to each author<br>• The full name of each funder<br>• URL of each funder website<br>• Did the sponsors or funders play any role in the study design, data collection and analysis, decision to publish, or preparation of the manuscript?<br>• **NO** - Include this sentence at the end of your statement: *The funders had no role in study design, data collection and analysis, decision to publish, or preparation of the manuscript.*<br>• **YES** - Specify the role(s) played.<br><br>* typeset	
**Competing Interests**<br><br>Use the instructions below to enter a competing interest statement for this submission. On behalf of all authors, disclose any competing interests that could be perceived to bias this work—acknowledging all financial support and any other relevant financial or non-financial competing interests.<br><br>This statement is required for submission and **will appear in the published article** if the submission is accepted. Please make sure it is accurate and that any funding sources listed in your Funding Information later in the submission form are also declared in your Financial Disclosure statement.<br><br>View published research articles from *PLOS ONE* for specific examples.	The authors have declared that no competing interests exist.

**NO authors have competing interests**<br><br>Enter: *The authors have declared that no competing interests exist.*<br><br>**Authors with competing interests**<br><br>Enter competing interest details beginning with this statement:<br><br>*I have read the journal's policy and the authors of this manuscript have the following competing interests: [insert competing interests here]*<br><br>* typeset	
**Ethics Statement**<br><br>Enter an ethics statement for this submission. This statement is required if the study involved:<br><br>• Human participants<br>• Human specimens or tissue<br>• Vertebrate animals or cephalopods<br>• Vertebrate embryos or tissues<br>• Field research<br><br>Write "N/A" if the submission does not require an ethics statement.<br><br>General guidance is provided below. Consult the submission guidelines for detailed instructions. **Make sure that all information entered here is included in the Methods section of the manuscript.**	Ethical clearance was obtained from Ethical Review committee of the University Hospital Bonn, and the Addis Ababa Regional Reference Laboratory. In addition, support letter was obtained from University Hospital Bonn, Institute of Hygiene and Public Health. The selected health facilities were informed about the study and a written consent was obtained. The collected data privacy and confidentiality were protected at all times of the data collection period. All participants' identifiers were removed during analysis once the data collection and quality assurance processes were over.

Powered by Editorial Manager® and ProduXion Manager® from Aries Systems Corporation

**Format for specific study types**<br><br>**Human Subject Research (involving human participants and/or tissue)**<br>• Give the name of the institutional review board or ethics committee that approved the study<br>• Include the approval number and/or a statement indicating approval of this research<br>• Indicate the form of consent obtained (written/oral) or the reason that consent was not obtained (e.g. the data were analyzed anonymously)<br><br>**Animal Research (involving vertebrate animals, embryos or tissues)**<br>• Provide the name of the Institutional Animal Care and Use Committee (IACUC) or other relevant ethics board that reviewed the study protocol, and indicate whether they approved this research or granted a formal waiver of ethical approval<br>• Include an approval number if one was obtained<br>• If the study involved *non-human primates*, add *additional details* about animal welfare and steps taken to ameliorate suffering<br>• If anesthesia, euthanasia, or any kind of animal sacrifice is part of the study, include briefly which substances and/or methods were applied<br><br>**Field Research**<br><br>Include the following details if this study involves the collection of plant, animal, or other materials from a natural setting:<br>• Field permit number<br>• Name of the institution or relevant body that granted permission	
**Data Availability**<br><br>Authors are required to make all data underlying the findings described fully available, without restriction, and from the time of publication. PLOS allows rare exceptions to address legal and ethical concerns. See the PLOS Data Policy and FAQ for detailed information.	Yes - all data are fully available without restriction

Powered by Editorial Manager® and ProduXion Manager® from Aries Systems Corporation

A Data Availability Statement describing where the data can be found is required at submission. Your answers to this question constitute the Data Availability Statement and **will be published in the article**, if accepted.<br><br>**Important:** Stating 'data available on request from the author' is not sufficient. If your data are only available upon request, select 'No' for the first question and explain your exceptional situation in the text box.<br><br>Do the authors confirm that all data underlying the findings described in their manuscript are fully available without restriction?	
**Describe where the data may be found in full sentences. If you are copying our sample text, replace any instances of XXX with the appropriate details.**<br><br>• If the data are **held or will be held in a public repository**, include URLs, accession numbers or DOIs. If this information will only be available after acceptance, indicate this by ticking the box below. For example: *All XXX files are available from the XXX database (accession number(s) XXX, XXX.).*<br>• If the data are all contained **within the manuscript and/or Supporting Information files**, enter the following: *All relevant data are within the manuscript and its Supporting Information files.*<br>• If neither of these applies but you are able to provide **details of access elsewhere**, with or without limitations, please do so. For example:<br><br>*Data cannot be shared publicly because of [XXX]. Data are available from the XXX Institutional Data Access / Ethics Committee (contact via XXX) for researchers who meet the criteria for access to confidential data.*<br><br>*The data underlying the results presented in the study are available from (include the name of the third party*	All relevant data are within the manuscript and its Supporting Information files.

and contact information or URL).<br>• This text is appropriate if the data are owned by a third party and authors do not have permission to share the data.<br><br>* typeset	
Additional data availability information:	

Powered by Editorial Manager® and ProduXion Manager® from Aries Systems Corporation

Manuscript Click here to access/download;Manuscript;Manuscript.doc

Impact of GeneXpert on TB diagnosis and anti-TB treatment outcome at health facilities in Addis Ababa, Ethiopia: In the post-MDG years

Desalegn Addise[1,2*], Laura E. Layland[2], Achim Hoerauf[2,3&], Biniam Wondale[4]

[1] Ethiopian Public Health Institute, Addis Ababa, Ethiopia

[2] Institute of Medical Microbiology, Immunology and Parasitology, University Hospital Bonn, Bonn, Germany

[3] German Center for Infection Research (DZIF), partner site Bonn-Cologne, Germany

[4] Department of Biology, Arba Minch University, Arba Minch, Ethiopia

*Corresponding author: Email: desalegnaddise@gmail.com

Abstract

Background: GeneXpert is an effective and rapid molecular system used for tuberculosis (TB) diagnosis. It is expected to improve the detection rate and treatment outcome to meet the sustainable development goal (SDG) and End TB strategy target set for 2030. This study aimed to evaluate the impact of GeneXpert on diagnosis and anti-TB treatment outcomes in the post-millennium development goals (MDGs) in the capital city of Ethiopia. Hence, the global priority indicator based on the End TB Strategy for TB treatment success rate was met early in 2018 in Addis Ababa, Ethiopia, which is anticipated to be met by 2025.

Method: A retrospective health facilities-based study was conducted in Addis Ababa, Ethiopia. Records of all TB cases diagnosed and treated in selected health facilities from January 2015 to December 2018 were reviewed and included in the study. Data analysis of descriptive and inferential statistics was conducted using SPSS version 20.

Result: The reviewed records have shown that a total of 45,158 presumptive pulmonary TB (PTB) cases had accessed TB diagnosis services. Of which, 28.9% (13072/45158) were tested by AFB microscopy and 71.1% (32086/45158) were tested by GeneXpert. In the study period, the coverage of Xpert MTB/RIF testing was increased to 94.9% in 2018 compared to 1.6% in 2015. The number of presumptive PTB cases, who tested with the GeneXpert system compared to smear microscopy showed a significant increase. The odds of positivity were detected in males compared to females. The odds of detecting TB cases were much higher among study participants aged 15-44 years compared to younger than 15 years. Treatment success rate showed a relative improvement each year between 2015 and 2018 with a mean of 92.6%. Reduced odds of treatment successes were observed in age categories older than 35 years, and in TB/HIV co-infected patients. Increased odds of treatment successes were reported in the years between 2016 and 2018 compared to 2015.

Conclusion: Scaling up the Xpert MTB/RIF assay as a point-of-care test for presumptive TB cases in resource-limited settings would have a significant impact to meet the SDG and End TB strategy both in TB detection and treatment success rates.

Keywords: Tuberculosis, Treatment outcomes, GeneXpert, MDG, SDG, Addis Ababa, Ethiopia

Background

Tuberculosis (TB) is a chronic infectious disease mainly caused by *Mycobacterium tuberculosis* that affects primarily the lung. There were an estimated 10 million TB cases in 2019. Of which, there were 206,030 multi-drug resistant (MDR) including rifampicin resistance (RR), and 12,350 laboratory-confirmed extensively drug resistance (XDR) TB cases. About 1.4 million deaths happened due to TB [1].

In sub-Saharan African countries, the rapid increase in drug-resistance TB is a challenge [2]. The Xpert MTB/RIF assay is a solution to detect *M. tuberculosis* including RR cases at the same time [3,4]. In sputum samples with negative smear results, the Xpert MTB/RIF assay has a pooled sensitivity of 60.6-77% and specificity of 98.8% [5]. Due to its improved sensitivity and specificity over smear microscopy for the diagnosis of TB, the Xpert MTB/RIF assay is promised to improve the anti-TB treatment outcome [6–9]. Xpert MTB/RIF assay reduces the anti-TB treatment initiation time by around four weeks for patients with paucibacillary for *M. tuberculosis* [6,10]. With these conditions, World Health Organization (WHO) endorsed and recommended the use of the GeneXpert system at the point of care for the diagnosis of TB [11].

Proper diagnosis and treatment of TB are crucial for the effort towards meeting the sustainable development goal (SDG) and End TB strategy targets set for 2035. Ethiopia achieved the TB-related Millennium Development Goals (MDGs) with a reduction of deaths due to TB by 75% and the TB incidence rate by more than 50% in 2015 compared to 1990 [2]. The GeneXpert instruments were introduced and have been in use in Ethiopia since 2012 [12]. They are used to diagnose only selected presumptive TB cases (presumptive DR-TB, HIV positive, children, and extrapulmonary TB), while the remaining cases with low-risk for DR-TB, HIV negative, and adult are diagnosed by sputum-smear microscopy [13]. Sputum-smear microscopy is a first-line test despite having a very poor sensitivity (25-75% compared to culture) and being unable to detect drug-resistant strains [14]. Since 2017, the GeneXpert system is in use to diagnose all presumptive TB cases presented with signs and symptoms of TB at any given health facility in Addis Ababa. However, the impact of Xpert MTB/RIF assay in TB diagnosis and treatment outcome in health facilities of Addis Ababa is not evaluated. Therefore, this study aimed to review the available records at the selected

health facilities in Addis Ababa, Ethiopia, and to evaluate the impacts of Xpert MTB/RIF assay on the number of TB cases detected and successes in anti-TB treatment outcome.

Methods

Study setting

The study was conducted in 19 public health facilities, which provide TB diagnosis services by the Xpert MTB/RIF assay in Addis Ababa, Ethiopia. Addis Ababa is the largest and capital city covering an area of 540 km^2 with an estimated population of 3,384,569 according to the 2007 census (World population review 2020). The city administration is divided into 10 sub-cities.

The government health infrastructure of Addis Ababa includes hospitals, health centers, and Addis Ababa Public Health Research and Emergency Management (AAPHREM) hereafter we call Addis Ababa Regional Reference Laboratory (A.A. Ref. Lab).

Study design, data collection, and ethical review

A retrospective health facility-based study was conducted in the capital city of Ethiopia by reviewing the laboratory TB registry logbook and TB unit register. In Addis Ababa, the Xpert MTB/RIF testing service is provided in five hospitals, 18 health centers, and one regional reference laboratory. The type of methods used for TB diagnosis and the number of presumptive pulmonary TB cases tested in the study period between January 2015 and December 2018 were recorded from the laboratory TB registry logbook. All tests for all age groups of presumptive pulmonary TB cases were done using sputa as a sample source. All TB cases on anti-TB treatment, which were registered in the respective health facilities' TB unit register during the study period, were included in the study. Out of 24 health facilities, which provided Xpert MTB/RIF testing services, five health facilities were excluded from the study. Of the five excluded health facilities, four health facilities did not document TB registry logbooks and one health facility refused to participate in the study. Additional two health facilities were excluded from treatment outcome analysis since they only provided test service but not DOTs service. Hence, the laboratory data were collected from 19 health facilities whereas anti-TB treatment outcome data were collected from 17 health facilities.

Demographic and clinical data of TB cases were reviewed and recorded into Microsoft Excel by a data clerk. The correctness of the recorded data was cross-checked by the principal investigator.

Ethical clearance was obtained from the Ethical Review Committee of the University Hospital Bonn and the Addis Ababa Regional Reference Laboratory. In addition, a support letter was obtained from the University Hospital Bonn, Institute of Hygiene and Public Health. The selected health facilities were informed about the study and written consent was obtained. The collected data privacy and confidentiality were protected at all times during the data collection period. All participants' identifiers were removed during analysis once the data collection and quality assurance processes were completed.

Operational definitions:

The following treatment outcomes are defined as follows per WHO guideline 2013.

Cured – A pulmonary TB patient with bacteriologically confirmed TB at the beginning of treatment who was smear- or culture-negative in the last month of treatment and on at least one previous occasion.

Treatment completed – A TB patient who completed treatment without evidence of failure BUT with no record to show that sputum smear or culture results in the last month of treatment and on at least one previous occasion were negative, either because tests were not done or because results are unavailable.

Treatment failed – A TB patient whose sputum smear or culture is positive at month 5 or later during treatment.

Died – A TB patient who dies for any reason before starting or during the course of treatment.

Loss to follow-up – A TB patient who did not start treatment or whose treatment was interrupted for two consecutive months or more.

Not evaluated - A TB patient for whom no treatment outcome is assigned. This includes cases "transferred out" to another treatment unit as well as cases for whom the treatment outcome is unknown to the reporting unit.

Transfer-in - patients have been transferred from another TB register to continue treatment.

Successful treatment outcome – the sum of cured and treatment completed.

Data analysis and statistics

The data were primarily recorded in Microsoft Excel and then exported into SPSS Version 20.0 software (SPSS INC, Chicago, IL, USA) for analysis. The findings were presented using descriptive statistics. Bivariate and multivariate logistic regression models were also used to evaluate the association of independent variables with dependent variables. The level of significance was set at $P \leq 0.05$.

Results

Trends of presumptive pulmonary TB cases diagnosis from smear microscopy to GeneXpert system

The reviewed records have shown that a total of 45,158 presumptive pulmonary TB cases had accessed TB diagnosis services. Of the total presumptive pulmonary TB cases in the study period, 28.9% (13072/45158) were tested by acid-fast bacilli (AFB) microscopy whereas 71.1% (32086/45158) were tested by GeneXpert system Table 1. Xpert MTB/RIF testing coverage increased during the study period from 1.6% in 2015 to 94.9% in 2018, despite a reduction in AFB microscopy utilization from 98.4% in 2015 to 5.1% in 2018.

Table 1. Uptake of GeneXpert system compared to smear microscopy for TB diagnosis at Addis Ababa, Ethiopia, between 2015 and 2018.

Year	Method of Diagnosis		Total N (%)
	Microscopy N (%)	GeneXpert system N (%)	
2015	4560 (98.4)	75 (1.6)	4635 (10.3)
2016	5679 (61.2)	3597 (38.8)	9276 (20.5)
2017	1931 (14.1)	11744 (85.9)	13675 (30.3)
2018	902 (5.1)	16670 (94.9)	17572 (38.9)
Total	13072 (28.9)	32086 (71.1)	45158 (100.0)

TB positivity and associated factors within presumptive TB cases

After adjusting factors associated with TB case detection, the GeneXpert system had higher odds of detecting TB cases (AOR: 4.78, 95 % CI: 4.13 – 5.53) compared to smear microscopy. Presumptive TB cases were more likely to be diagnosed with TB if they were male (AOR: 1.45, 95% CI: 1.36–1.54) compared to female cases. The odds of detecting TB cases increased among study participants aged 15-44 years compared to study participants younger than 15 years Table 2.

Table 2. Factors associated with the positive results of presumptive TB cases in Addis Ababa, Ethiopia between 2015 and 2018

Variable	Total N (%)	Positive case N (%)	**COR (95% CI)**	**AOR (95% CI)**	**P-value**
Sex[a]					
F	22882 (50.7)	2081 (9.1)	1	1	
M	22242 (49.3)	2694 (12.1)	**1.38 (1.30 – 1.46)**	**1.45 (1.36 – 1.54)**	<0.001
Age[b]					
0-14	2309 (5.1)	113 (4.9)	1	1	
15-24	8359 (18.6)	1273 (15.2)	**3.49 (2.86 – 4.26)**	**3.72 (3.05 – 4.55)**	<0.001
25-34	11769 (26.2)	1582 (13.4)	**3.02 (2.48 – 3.67)**	**3.03 (2.48 – 3.69)**	<0.001
35-44	8075 (18.0)	822 (10.2)	**2.20 (1.80 – 2.70)**	**2.08 (1.69 – 2.55)**	<0.001
>=45	14436 (32.1)	809 (5.6)	1.15 (0.94 – 1.41)	1.11 (0.91 – 1.36)	0.313
Methods of Dx					
Microscope	13072 (28.9)	846 (6.5)	1	1	
GeneXpert	32086 (71.1)	3963 (12.4)	**2.04 (1.89 – 2.20)**	**4.78 (4.13 – 5.53)**	<0.001
Year of Dx					
2015	4635 (10.3)	455 (9.8)	1	1	
2016	9276 (20.5)	1167 (12.6)	**1.32 (1.18 – 1.48)**	**0.72 (0.62 – 0.83)**	<0.001
2017	13675 (30.3)	1525 (11.2)	**1.15 (1.03 – 1.29)**	**0.37 (0.31 – 0.44)**	<0.001
2018	17572 (38.9)	1662 (9.5)	0.96 (0.86 – 1.07)	**0.28 (0.24 – 0.34)**	<0.001
Sub-cities					
Addis Ketema	3564 (7.9)	284 (8.0)	1	1	
Kolfe Keranyo	8101 (17.9)	744 (9.2)	**1.17 (1.01 – 1.35)**	**1.53 (1.31 – 1.77)**	<0.001
Bole	1645 (3.6)	194 (11.8)	**1.54 (1.27 – 1.87)**	**1.43 (1.17 – 1.75)**	<0.001
Akaki-Kality	4759 (10.5)	524 (11.0)	**1.43 (1.23 – 1.66)**	**1.59 (1.36 – 1.86)**	<0.001
Yeka	7980 (17.7)	683 (8.6)	1.08 (0.94 – 1.25)	**1.23 (1.06 – 1.44)**	0.006
Arada	4897 (10.8)	470 (9.6)	**1.23 (1.05 – 1.43)**	**1.41 (1.20 – 1.65)**	<0.001
Nifas Silk	7028 (15.6)	710 (10.1)	**1.30 (1.12 – 1.50)**	**1.48 (1.28 – 1.72)**	<0.001
Kirkos	1704 (3.8)	147 (8.6)	1.09 (0.89 – 1.34)	**1.32 (1.06 – 1.64)**	0.013
A.A. Ref. Lab*	5480 (12.1)	1053 (19.2)	**2.75 (2.39 – 3.16)**	**1.42 (1.20 – 1.69)**	<0.001

[a]- of the total 34 participants had no sex information; [b]- of the total 210 participants had no age information; * stands for Addis Ababa Regional Reference Laboratory.

Demographics and clinical characteristics of study participants on anti-TB treatment

The total numbers of patients who received anti-TB treatment in 17 health facilities were 8446 including extrapulmonary TB (EPTB) cases. Table 3 details the demographic and clinical characteristics of TB patients who received anti-TB treatment during the study periods.

The majority, 77% (6504/8446) of TB patients were in the age group 15-44 years. Male patients 51.7% (4365/8446), who received treatment in the study period were slightly higher

than females. The percentages of patients with no anti-TB treatment history, re-treatment, and transfer-in cases were 86.3% (7282/8446), 9.4% (797/8446), and 4.3% (367/8446), respectively. TB/HIV co-infection was 20.4% (1723/8446). TB cases in the study period were PTBN 26.7% (2252/8446), PTBP 29.6% (2500/8446), and EPTB 43.7% (3694/8446).

Table 3. Demographics and clinical characteristics of TB patients who received anti-TB treatment in Addis Ababa, Ethiopia between 2015 and 2018.

**Variables**	**TB Types in Number (%)**			**Total N (%)**
	**PTBN**	**PTBP**	**EPTB**	
Sex				
F	992 (44.0)	1097 (43.9)	1992 (53.9)	4081 (48.3)
M	1260 (56.0)	1403 (56.1)	1702 (46.1)	4365 (51.7)
Age				
< 15	84 (3.7)	53 (2.1)	178 (4.8)	315 (3.7)
15-24	533 (23.7)	763 (30.5)	1012 (27.4)	2308 (27.3)
25-34	596 (26.5)	895 (35.8)	1213 (32.9)	2704 (32.0)
35-44	431 (19.1)	418 (16.7)	643 (17.4)	1492 (17.7)
45-54	257 (11.4)	202 (8.1)	283 (7.7)	742 (8.8)
>=55	351 (15.6)	169 (6.8)	359 (9.7)	879 (10.4)
Unknown	0 (0.0)	0 (0.0)	6 (0.1)	6 (0.1)
TB History Category				
New	1871 (83.1)	2134 (85.4)	3277 (88.7)	7282 (86.3)
Re-treatment	282 (12.5)	238 (9.5)	277 (7.5)	797 (9.4)
Transfer in	99 (4.4)	128 (5.1)	140 (3.8)	367 (4.3)
HIV Status of TB Cases				
Non-infected	1627 (72.2)	1920 (76.8)	2812 (76.1)	6359 (75.3)
Infected	532 (23.6)	476 (19.0)	715 (19.4)	1723 (20.4)
Unknown	93 (4.1)	104 (4.2)	167 (4.5)	364 (4.3)
Year of Registration				
2015	726 (32.2)	585 (23.4)	976 (26.4)	2287 (27.1)
2016	668 (29.7)	536 (21.4)	946 (25.6)	2150 (25.5)
2017	458 (20.3)	636 (25.4)	874 (23.7)	1968 (23.3)
2018	400 (17.8)	743 (29.7)	898 (24.3)	2041 (24.2)
Sub-cities in Addis Ababa				
Addis Ketema	147 (6.5)	144 (5.8)	236 (6.4)	527 (6.2)
Kolfe Keranyo	610 (27.1)	720 (28.8)	1003 (27.2)	2333 (27.6)
Bole	122 (5.4)	148 (5.9)	205 (5.5)	475 (5.6)
Akaki-Kaliti	176 (7.8)	196 (7.8)	229 (6.2)	601 (7.1)
Yeka	606 (26.9)	506 (20.2)	753 (20.4)	1865 (22.1)
Arada	27 (1.2)	37 (1.5)	83 (2.2)	147 (1.7)
Nefas Silk	518 (23.0)	701 (28.0)	984 (26.6)	2203 (26.1)
Kirkos	46 (2.0)	48 (1.9)	201 (5.4)	295 (3.5)

Total	2252 (26.7)	2500 (29.6)	3694 (43.7)	8446 (100)

PTBN - Pulmonary TB clinically; **PTBP** - Pulmonary TB bacteriologically; **EPTB** - Extra-pulmonary TB

Treatment outcome

Overall, 7478 out of 8080 TB patients (92.6%) had successful treatment outcomes. Of these, 27.3% (2205/8080) patients had been cured and 65.3% (5273/8080) patients had completed treatment. HIV-infected TB patients had a lower rate of treatment success 86.8% (1416/1632) compared to non-infected ones 94.2% (5765/6121). Treatment outcomes after 2015 showed relative improvement Table 4.

Table 4. Treatment outcomes of TB patients at Addis Ababa, Ethiopia between 2015 and 2018.

Variables	Treatment Outcome[a] in Number (%)					Total N (%)	P-value
	Successful		Unsuccessful				
	Cured	T. Completed	Died	T. Failed	Loss to Follow-Up		
Sex							
F	965 (24.7)	2676 (68.6)	22 (0.6)	196 (5.0)	44 (1.1)	3903 (48.3)	< 0.001
M	1240 (29.7)	2597 (62.2)	26 (0.6)	222 (5.3)	92 (2.2)	4177 (51.7)	
Age Category in years							
< 15	48 (15.5)	249 (80.6)	1 (0.3)	8 (2.6)	3 (1.0)	309 (3.8)	< 0.001
15-24	689 (30.9)	1439 (64.5)	20 (0.9)	44 (2.0)	38 (1.7)	2230 (27.6)	
25-34	789 (30.5)	1662 (64.3)	11 (0.4)	86 (3.3)	37 (1.4)	2585 (32.0)	
35-44	366 (25.7)	922 (64.7)	9 (0.6)	104 (7.3)	24 (1.7)	1425 (17.6)	
45-54	175 (24.9)	444 (63.2)	2 (0.3)	66 (9.4)	15 (2.1)	702 (8.7)	
>=55	138 (16.8)	552 (67.1)	5 (0.6)	110 (13.4)	18 (2.2)	823 (10.2)	
Unknown	0 (0.0)	5 (83.3)	0 (0.0)	0 (0.0)	1 (16.7)	6 (0.1)	
TB History Category							
New	1916 (27.3)	4604 (65.6)	45 (0.6)	344 (4.9)	109 (1.6)	7018 (86.9)	0.005
Re-treatment	189 (25.9)	468 (64.0)	2 (0.3)	56 (7.7)	16 (2.2)	731 (9.0)	
Transfer in	100 (30.2)	201 (60.7)	1 (0.3)	18 (5.4)	11 (3.3)	331 (4.1)	
TB Type							
PTBN	NA	1994 (91.6)	7 (0.3)	140 (6.4)	35 (1.6)	2176 (26.9)	< 0.001
PTBP	2205 (92.9)	0 (0.0)	29 (1.2)	82 (3.5)	58 (2.4)	2374 (29.4)	
EPTB	NA	3279 (92.9)	12 (0.3)	196 (5.6)	43 (1.2)	3530 (43.7)	
HIV Status of TB Cases							

Non-infected	1738 (28.4)	4027 (65.8)	38 (0.6)	211 (3.4)	107 (1.7)	6121 (75.8)	< 0.001
Infected	384 (23.5)	1032 (63.2)	9 (0.6)	184 (11.3)	23 (1.4)	1632 (20.2)	
Unknown	83 (25.4)	214 (65.4)	1 (0.3)	23 (7.0)	6 (1.8)	327 (4.0)	
Year of Registration							
2015	488 (22.8)	1452 (67.9)	17 (0.8)	134 (6.3)	49 (2.3)	2140 (26.5)	<0.001
2016	469 (22.7)	1453 (70.3)	11 (0.5)	100 (4.8)	35 (1.7)	2068 (25.6)	
2017	575 (30.0)	1215 (63.4)	8 (0.4)	89 (4.6)	29 (1.5)	1916 (23.7)	
2018	673 (34.4)	1153 (58.9)	12 (0.6)	95 (4.9)	23 (1.2)	1956 (24.2)	
Sub-cities							
Addis Ketema	119 (23.8)	337 (67.3)	5 (1.0)	29 (5.8)	11 (2.2)	501 (6.2)	<0.001
Kolfe Keranyo	636 (28.2)	1459 (64.7)	9 (0.4)	119 (5.3)	31 (1.4)	2254 (27.9)	
Bole	141 (30.5)	298 (64.4)	2 (0.4)	22 (4.8)	0 (0.0)	463 (5.7)	
Akaki-Kaliti	175 (29.6)	369 (62.4)	8 (1.4)	29 (4.9)	10 (1.7)	591 (7.3)	
Yeka	451 (25.3)	1195 (66.9)	11 (0.6)	99 (5.5)	30 (1.7)	1786 (22.1)	
Arada	34 (24.8)	91 (66.4)	0 (0.0)	12 (8.8)	0 (0.0)	137 (1.7)	
Nefas Silk	612 (29.1)	1341 (63.8)	13 (0.6)	88 (4.2)	47 (2.2)	2101 (26.0)	
Kirkos	37 (15.0)	183 (74.1)	0 (0.0)	20 (8.1)	7 (2.8)	247 (3.1)	
**Total**	**2205 (27.3)**	**5273 (65.3)**	**48 (0.6)**	**418 (5.2)**	**136 (1.7)**	**8080 (100.0)**	

[a] TB patient whose treatment outcome is not evaluated/recorded (301) and RR-TB cases transferred to MDR Treatment initiating centers (65) were excluded during analysis. NA- Not applicable; T. completed- treatment completed; T. failed- treatment failed

Treatment success and associated factors

After adjusting factors associated with treatment outcome, age categories 35-44 (AOR: 0.49, 95% CI: 0.27 – 0.90), 45-54 (AOR: 0.36, 95% CI: 0.19 – 0.67), > =55 (AOR: 0.20, 95% CI: 0.11 – 0.37) and TB/HIV co-infected patients (AOR: 0.44 95% CI: 0.36 – 0.53) were predicted with lower odds of successful treatment outcome. Whereas patients receiving treatment at Amoraw Health Center in Bole Sub-city had a better treatment success rate (AOR: 1.73, 95% CI: 1.03 – 2.91) compared to Addis Ketema Health Center in Addis Ketema Sub-city Table 5.

Table 5. Factors associated with successful treatment outcomes of TB patients in Addis Ababa, Ethiopia between 2015 and 2018.

Variables	Total N (%)	Treatment success N (%)	COR (95% CI)	AOR (95% CI)	p-value
Sex					
F	3903 (48.3)	3641 (93.3)	1	1	
M	4177 (51.7)	3837 (91.9)	**0.81 (0.69 – 0.96)**	0.90 (0.76 – 1.08)	0.268
Age					
0-14	309 (3.8)	297 (96.1)	1	1	
15-24	2230 (27.6)	2128 (95.4)	0.84 (0.46 – 1.55)	0.76 (0.41 – 1.40)	0.378
25-34	2585 (32.0)	2451 (94.8)	0.74 (0.40 – 1.35)	0.77 (0.42 – 1.42)	0.407
35-44	1425 (17.6)	1288 (90.4)	**0.38 (0.21 – 0.69)**	**0.49 (0.27 – 0.90)**	**0.021**
45-54	702 (8.7)	619 (88.2)	**0.30 (0.16 – 0.56)**	**0.36 (0.19 – 0.67)**	**0.001**
>=55	823 (10.2)	690 (83.8)	**0.21 (0.11 – 0.38)**	**0.20 (0.11 – 0.37)**	**<0.001**
Unknown	6 (0.1)	5 (83.3)	0.20 (0.02 – 1.87)	0.23 (0.02 – 2.20)	0.201
TB History Category					
New	7018 (86.9)	6520 (92.9)	1	1	
Re-treatment	731 (9.0)	657 (89.9)	**0.68 (0.52 – 0.88)**	0.90 (0.69 – 1.18)	0.451
Transfer in	331 (4.1)	301 (90.9)	0.77 (0.52 – 1.13)	0.78 (0.53 – 1.17)	0.229
TB Type					
PTBN	2176 (26.9)	1994 (91.6)	1	1	
PTBP	2374 (29.4)	2205 (92.9)	1.19 (0.96 – 1.48)	0.93 (0.74 – 1.16)	0.504
EPTB	3530 (43.7)	3279 (92.9)	1.19 (0.98 – 1.46)	0.98 (0.80 – 1.21)	0.863
HIV Status of TB Cases					
Non-infected	6121 (75.8)	5765 (94.2)	1	1	
Infected	1632 (20.2)	1416 (86.8)	**0.41 (0.34 – 0.48)**	**0.44 (0.36 – 0.53)**	**<0.001**
Unknown	327 (4.0)	297 (90.8)	**0.61 (0.41 – 0.90)**	**0.60 (0.40 – 0.91)**	**0.016**
Year of Registration					
2015	2140 (26.5)	1940 (90.7)	1	1	
2016	2068 (25.6)	1922 (92.9)	**1.36 (1.09 – 1.70)**	**1.39 (1.10 – 1.75)**	**0.006**
2017	1916 (23.7)	1790 (93.4)	**1.47 (1.16 – 1.85)**	**1.42 (1.12 - 1.80)**	**0.004**

2018	1956 (24.2)	1826 (93.4)	**1.45 (1.15 – 1.82)**	**1.43 (1.13 – 1.81)**	**0.003**
Sub-cities					
Addis Ketema	501 (6.2)	456 (91.0)	1	1	
Kolfe Keranyo	2254 (27.9)	2095 (92.9)	1.30 (0.92 – 1.84)	1.30 (0.91 – 1.85)	0.148
Bole	463 (5.7)	439 (94.8)	**1.81 (1.08 – 3.01)**	**1.73 (1.03 – 2.91)**	**0.039**
Akaki-Kaliti	591 (7.3)	544 (92.0)	1.14 (0.75 – 1.75)	1.21 (0.78 – 1.88)	0.387
Yeka	1731 (21.4)	1595 (92.1)	1.16 (0.82 – 1.65)	1.21 (0.85 – 1.75)	0.289
Arada	192 (2.4)	176 (91.7)	1.09 (0.60 – 1.97)	1.38 (0.75 – 2.55)	0.299
Nefas Silk	2101 (26.0)	1953 (93.0)	1.30 (0.92 – 1.85)	1.28 (0.90 – 1.84)	0.171
Kirkos	247 (3.1)	220 (89.1)	0.80 (0.49 – 1.33)	1.13 (0.67 – 1.92)	0.650

Bold- values, which show significance (< 0.05); COR- crude odd ratio; AOR- adjusted odd ratio

4. Discussion

Early diagnosis of presumptive TB cases has great importance for improving the treatment outcome and reducing the risk of transmitting the disease to others. Xpert MTB/RIF assay has more sensitivity than smear microscopy in detecting TB cases [15–20]. This study was to evaluate the TB diagnosis and treatment outcome in health facilities of Addis Ababa, Ethiopia after the introduction of the GeneXpert system. The study showed that the trends of shifting pulmonary TB diagnosis methods from smear microscopy to GeneXpert system brought an improvement in case detection and success rate of treatment outcomes.

The introduction of Xpert MTB/RIF assay in Addis Ababa increased the number of identified TB cases each year to almost four-fold in 2018 with reference to 2015. This might be due to the high sensitivity of Xpert MTB/RIF assay in *M. tuberculosis* detection [15–20]. This finding has been supported by the logistic regression analysis result of the current study. Presumptive TB cases diagnosed with GeneXpert had 4.78 odds of being TB positive compared to those were diagnosed by microscopy. This finding was consistent with the previous studies performed on the advanced diagnostic performance of the GeneXpert system over smear microscopy that revealed a higher TB cases detection [17,21–26].

Male patients who were diagnosed with TB had higher odds (AOR: 1.45, 95% CI: 1.36–1.54) of being TB positive compared to females. This finding is concordant with findings from the 2016 Global Burden of Diseases and different studies conducted in different countries, particularly in low and middle-income countries [27–33]. Such sex differences might attribute to biological TB susceptibility differences between males and females [34], but as indicated by Manish Gupta the underlying genetic mechanism is not clearly defined [35]. The economically productive age group, between 15 and 44 years, was 62.8% of patients diagnosed for TB and they had higher odds of being positive compared to the age group less than 14 years. This might be due to the travel history of the economically productive age group far from their home looking for education, jobs, and social interactions. These might increase the chance of being in contact with infected individuals. The other factor associated with children less than 14 years of age might be the sputum quality that is children are more unlikely to produce good-quality sputum [36].

The male patients enrolled for DOTs service in the study period were 51.7%, which is comparable to reports from Addis Ababa 51% [37]. It is also found lower than findings from different parts of Ethiopia; 53.8% in Asella Teaching Hospital [38], 58% in Mekele [39], while slightly higher than 46.8% in Addis Ababa [40]. Among the total TB cases that received anti-TB treatment 20.4% of TB cases were HIV-positive. This was slightly higher than the HIV-positive rate reported in Northern Ethiopia 18.8% [39] but lower than among notified TB cases in 2016 in Addis Ababa 28% [41].

Among pulmonary TB-positive cases, 92.9% were cured. Of all TB types, 92.6% of TB patients had successful treatment outcomes. The mean treatment success rate in this study was higher than treatment success rates reported in districts of Southwestern (88%), Asela Teaching Hospital (81.7%), and in the refugee camps (75.9%) in Ethiopia [38,42,43]. But relatively lower than the treatment outcome notified for new TB cases in 2017 in Ethiopia 96% [44]. This finding has revealed that a 30% decrease in death rate was seen in 2018 among patients on anti-TB treatment compared with 2015. The increase in trends of treatment success between consecutive years showed a relative improvement in each year between 2015 and 2018. The global priority indicator and target for all countries were planned to reach a 90% treatment success rate at the latest 2025 [45]. In this regard, the End TB Strategy towards the TB treatment success rate was met early in 2018 in Addis Ababa, Ethiopia.

The odds of treatment outcome in age groups and TB/HIV co-infection in this study was in agreement with findings from Addis Ababa, Asella Teaching Hospital, and Debre Tabor General Hospital [37,38,46]. TB patients with ages >= 35 years and being co-infected with HIV were likely to have a lower treatment success rate. In elder age group, this might be due to non-adherence to TB treatment as a result of possible factors such as family responsibility and being busy with socio-economic activities [47]. In TB/HIV co-infected patients, it might be due to the presence of pills burden when both TB and ART drugs were taken which leads to drug-drug interactions, overlapping drug toxicities, and immune reconstitution syndrome [48]. Patients receiving anti-TB treatment at Amoraw HC in Bole Sub-city were predicted to have higher positive treatment outcomes compared to Addis Ketema HC in Addis Ketema Sub-city. This might be due to patients at Bole Sub-city having relatively good socio-economic and housing status compared to Addis Ketema Sub-city [49,50].

This study was able to evaluate the significance of diagnosing presumptive TB cases with GeneXpert system over microscopy by including 45,158 presumptive PTB cases and the TB treatment outcome by assessing records of 8080 TB cases in the study area. However, it has the following limitations: it did not include bacteriologically confirmed PTB cases which were diagnosed in two health facilities without TB treatment unit in the treatment outcome analysis; second due to the nature of retrospective secondary data, there were missing data like sex and age categories. However, as we have included more health facilities and a large number of TB cases in the study, the effect of missing data was considered not to be significant; third this study did not assess other factors or actions taken in health facilities to improve treatment outcome, which might have a positive role in treatment outcome improvement in addition to GeneXpert system. Lastly, this study lacks comorbidities other than HIV such as diabetes, hypertension, obesity, cancer etc which might have an effect on TB treatment outcome.

5. Conclusion

The introduction of the GeneXpert system in Addis Ababa, Ethiopia had increased the number of confirmed TB cases with odds of 4.78 compared to the smear microscopy. The success of treatment outcomes steadily improved each year in the study period. The overall success rate was 92.6%. Therefore, scaling up the Xpert MTB/RIF assay as a point-of-care test for presumptive TB cases in resource-limited settings would have a significant impact on meeting the SDG and End TB strategy both in TB detection and anti-TB treatment outcome.

Authors' contributions

DA was the primary researcher, conceived the study, designed, participated in data collection, conducted data analysis, and drafted the manuscript for publication. LEL, AH, and BW participated in the study design, data analysis, result interpretation, and reviewed the initial and final drafts of the manuscript. All authors read and approved the final manuscript.

Competing interests

The authors declare that they have no competing interests.

Acknowledgments

The authors would like to thank the Institutes of Hygiene and Public Health and of Medical Microbiology, Immunology and Parasitology, University Hospital Bonn and Addis Ababa Public Health Research and Emergency Management Ethical Review Office for reviewing the study protocol and writing a support letter for health facilities. We would like to thank Tewodros Daniel, Ashenafi Alemu, and Alem Mekonnen for their unreserved cooperation throughout the course of data collection. We are also grateful to health professionals working at the study sites because without their willingness, cooperation, and support it would not be possible to come up with these findings.

References

1. WHO (World Health Organization). Global Tuberculosis Report 2020. Geneva; 2020.
2. WHO (World Health Organization). Global Tuberculosis Report 2018. 2018.
3. Gesine Meyer-Rath, Lawrence Long, William Macleod, Ian Sanne, Kate Bistline, Wendy Stevens SR. The incremental cost of introducing Xpert® MTB/RIF into the South African national tuberculosis programme: Results of the National TB Cost Model 2011/12 - 2016/17. Johannesburg; 2016.
4. Raizada N, Sachdeva KS, Sreenivas A, Vadera B, Gupta RS, Parmar M, et al. Feasibility of Decentralised Deployment of Xpert MTB / RIF Test at Lower Level of Health System in India. 2014;9(2).
5. Rimal R, Shrestha D, Pyakurel S, Poudel R, Shrestha P, Rai KR, et al. Diagnostic performance of GeneXpert MTB/RIF in detecting MTB in smear-negative presumptive TB patients. BMC Infect Dis [Internet]. 2022 Dec 1 [cited 2023 Apr 7];22(1):1–7. Available from: https://bmcinfectdis.biomedcentral.com/articles/10.1186/s12879-022-07287-5
6. Kwak N, Choi SM, Lee J, Park YS, Lee C hoon, Lee S min, et al. Diagnostic Accuracy and Turnaround Time of the Xpert MTB / RIF Assay in Routine Clinical Practice. 2013;8(10):1–6.
7. Millman AJ, Dowdy DW, Miller CR, Brownell R, Metcalfe JZ, Cattamanchi A, et al. Rapid Molecular Testing for TB to Guide Respiratory Isolation in the U . S .: A Cost-Benefit Analysis. 2013;8(11):1–8.
8. Menzies NA, Cohen T, Lin H ho, Murray M, Salomon JA. Population Health Impact and Cost-Effectiveness of Tuberculosis Diagnosis with Xpert MTB / RIF : A Dynamic Simulation and Economic Evaluation. 2012;9(11).
9. Creswell J, Codlin AJ, Andre E, Micek MA, Bedru A, Carter EJ, et al. Results from early programmatic implementation of Xpert MTB / RIF testing in nine countries. 2014;

10. Omrani AS, Al-otaibi MF, Al-ateah SM, Al-onazi FM, Baig K, El-khizzi NA, et al. GeneXpert MTB / RIF Testing in the Management of Patients with Active Tuberculosis ; A Real Life Experience from Saudi Arabia. 2014;46(1):30–4.

11. WHO (World Health Organization), Programme GT. Automated Real-Time Nucleic Acid Amplification Technology for Rapid and Simultaneous Detection of Tuberculosis and Rifampicin Resistance: Xpert MTB/RIF Assay for the Diagnosis of Pulmonary and Extrapulmonary TB in Adults and Children: Policy update. World Heal Organ [Internet]. 2013 [cited 2020 Dec 26];1–79. Available from: www.who.int

12. Alemu A, Amare M, Ameni G, Kebede A, Beyene D, Yenew B, et al. Monitoring quality indicators for the Xpert MTB/RIF molecular assay in Ethiopia. PLoS One [Internet]. 2019 Nov 1 [cited 2023 Apr 6];14(11):e0225205. Available from: https://journals.plos.org/plosone/article?id=10.1371/journal.pone.0225205

13. FMoH Ethiopia. Implementation Guideline for GeneXpert MTB/RIF Assay in Ethiopia [Internet]. 2014 [cited 2023 Apr 6]. Available from: https://ephi.gov.et/wp-content/uploads/2014/04/Implementation-Guideline-for-GeneXpert-MTBRIF-Assay-in-Ethiopia-Nov-03-2....pdf

14. WHO, FMoH. Guidelines for Clinical and Programmatic Management of TB , TB / HIV , DR-TB and Leprosy in Ethiopia. 2021;7(August):1–249.

15. Maynard-Smith L, Larke N, Peters JA, Lawn SD. Diagnostic accuracy of the Xpert MTB/RIF assay for extrapulmonary and pulmonary tuberculosis when testing non-respiratory samples: A systematic review. BMC Infect Dis. 2014 Dec 31;14(1).

16. Sorsa A, Kaso M. Diagnostic performance of GeneXpert in tuberculosis–HIV co–infected patients at Asella Teaching and Referral Hospital, Southeastern Ethiopia: A cross sectional study. PLoS One [Internet]. 2021 Jan 1 [cited 2021 Sep 23];16(1). Available from: /pmc/articles/PMC7840185/

17. Habte D, Melese M, Hiruy N, Gashu Z, Jerene D, Moges F, et al. The additional yield of GeneXpert MTB/RIF test in the diagnosis of pulmonary tuberculosis among household contacts of smear positive TB cases. Int J Infect Dis [Internet]. 2016 Aug 1 [cited 2021 Mar 21];49:179–84. Available from: https://pubmed.ncbi.nlm.nih.gov/27401584/

18. Pinyopornpanish K, Chaiwarith R, Pantip C, Keawvichit R, Wongworapat K, Khamnoi P, et al. Comparison of Xpert MTB/RIF Assay and the Conventional Sputum Microscopy in Detecting Mycobacterium tuberculosis in Northern Thailand . Tuberc Res Treat. 2015;2015:1–6.

19. Steingart KR, Schiller I, Horne DJ, Pai M, Boehme CC, Dendukuri N. Xpert® MTB/RIF assay for pulmonary tuberculosis and rifampicin resistance in adults. Cochrane Database Syst Rev. 2014 Jan 21;2014(1).

20. Lange B, Khan P, Kalmambetova G, Al-Darraji HA, Alland D, Antonenka U, et al. Diagnostic accuracy of the Xpert® MTB/RIF cycle threshold level to predict smear positivity: A meta-analysis. Int J Tuberc Lung Dis. 2017 May 1;21(5):493–502.

21. Khadka P, Thapaliya J, Basnet RB, Ghimire GR, Amatya J, Rijal BP. Diagnosis of tuberculosis from smear-negative presumptive TB cases using Xpert MTB/Rif assay: A cross-sectional study from Nepal. BMC Infect Dis [Internet]. 2019 Dec 30 [cited 2021 Mar 21];19(1):1090. Available from: https://bmcinfectdis.biomedcentral.com/articles/10.1186/s12879-019-4728-2

22. Zahoor D, Farhana A, Kanth F, Bha M. Evaluation of smear microscopy and geneXpert for the rapid diagnosis of pulmonary and extrapulmonary tuberculosis in a tertiary care hospital in North India: a descriptive prospective study. Int J Res Med Sci [Internet]. 2018 Apr 25 [cited 2021 Mar 21];6(5):1756. Available from: www.msjonline.org

23. Neil M. Ampel M. Diagnosing TB with the Xpert Assay vs. AFB Smear Microscopy. NEJM J Watch [Internet]. 2019 Mar 25 [cited 2021 Mar 21];2019. Available from: https://www.jwatch.org/NA48799/2019/03/25/diagnosing-tb-with-xpert-assay-vs-afb-smear-microscopy

24. Orina GM, Ong’wen SA, Amolo S, Orindi OT, Oginga J. Comparative Study of Smear Microscopy, Gene Xpert and Culture and Sensitivity Assays in Detection of Mycobacterium tuberculosis on Sputum Samples among Tuberculosis Suspected Cases in Nyamira County Referral Hospital. 2017;

25. Taddese BD, Desalegn DM, Misganaw AS, Kitila KT, Hailu TK, Bika AT. Diagnostic

Performance of Xpert MTB/RIF Assay Versus Ziehl-Neelsen Method for the Diagnosis of Pulmonary Tuberculosis in Addis Ababa, Ethiopia. Open Microbiol J. 2018 Dec 13;12(1):390–6.

26. Arora D, Dhanashree B. Utility of smear microscopy and genexpert for the detection of mycobacterium tuberculosis in clinical samples. GERMS [Internet]. 2020 [cited 2021 Mar 4];10(2):81–7. Available from: /pmc/articles/PMC7330512/

27. Onifade DA, Bayer AM, Montoya R, Haro M, Alva J, Franco J, et al. Gender-related factors influencing tuberculosis control in shantytowns: A qualitative study. BMC Public Health [Internet]. 2010 Dec 29 [cited 2021 Mar 22];10(1):381. Available from: http://bmcpublichealth.biomedcentral.com/articles/10.1186/1471-2458-10-381

28. Diwan VK, Thorson A. Sex, gender, and tuberculosis [Internet]. Vol. 353, Lancet. Elsevier Limited; 1999 [cited 2021 Mar 22]. p. 1000–1. Available from: http://www.thelancet.com/article/S0140673699013185/fulltext

29. World Health Organization (WHO). Tuberculosis Gender in Research. 2005.

30. Yang W teng, Gounder CR, Akande T, Neve J walter De, Mcintire KN, Chandrasekhar A, et al. Barriers and Delays in Tuberculosis Diagnosis and Treatment Services : Does Gender Matter ? 2014;2014.

31. Thorson A, Hoa NP, Long NH, Allebeck P, Diwan VK. Do women with tuberculosis have a lower likelihood of getting diagnosed? Prevalence and case detection of sputum smear positive pulmonary TB, a population-based study from Vietnam. J Clin Epidemiol [Internet]. 2004 Apr [cited 2021 Mar 22];57(4):398–402. Available from: https://pubmed.ncbi.nlm.nih.gov/15135842/

32. Krishnan L, Akande T, Shankar A V, Mcintire KN, Gounder CR, Gupta A, et al. Gender-Related Barriers and Delays in Accessing Tuberculosis Diagnostic and Treatment Services : A Systematic Review of Qualitative Studies. 2014;2014.

33. Amare D, Deribe K, Dejene T, Tessema GA, Melaku YA, Lakew Y, et al. Tuberculosis Burden in Ethiopia from 1990 to 2016 : Evidence from the Global Burden of Diseases 2016 Study. 2018;

34. Neyrolles O, Quintana-Murci L. Sexual Inequality in Tuberculosis. PLOS Med [Internet]. 2009 Dec [cited 2022 Jun 16];6(12):e1000199. Available from: https://journals.plos.org/plosmedicine/article?id=10.1371/journal.pmed.1000199

35. Gupta M, Srikrishna G, Klein SL, Bishai WR. Genetic and hormonal mechanisms underlying sex-specific immune responses in tuberculosis. Trends Immunol. 2022 Aug 1;43(8):640–56.

36. Hare JD. Manual of Clinical Microbiology. Arch Pediatr Adolesc Med [Internet]. 1975 Jun 1 [cited 2021 Mar 20];129(6):754. Available from: http://archpedi.jamanetwork.com/article.aspx?doi=10.1001/archpedi.1975.02120430086036

37. Alem Y, Gebre-selassie S. Treatment Outcome of Tuberculosis Patients in Selected Health Centres in Addis Ababa : a Five Year Retrospective Study. 2017;1:5–12.

38. Tafess K, Tasew G, Mekit S, Beyen TK, Abera A, Solomon Sisay, et al. Treatment Outcomes of Tuberculosis at asella Teaching hospital , ethiopia : Ten Years ' retrospective aggregated Data. Front Med. 2018;5(February):1–9.

39. Abdulkader M, Aken I Van, Niguse S, Hailekiros H, Spigt M. Treatment outcomes and their trend among tuberculosis patients treated at peripheral health settings of Northern Ethiopia between 2009 and 2014 : a registry ‑ based retrospective analysis. BMC Res Notes [Internet]. 2019;10–4. Available from: https://doi.org/10.1186/s13104-019-4824-9

40. Getahun B, Ameni G, Medhin G, Biadgilign S. Original article Treatment outcome of tuberculosis patients under directly observed treatment in Addis Ababa , Ethiopia. Brazilian J Infect Dis [Internet]. 2013;17(5):521–8. Available from: http://dx.doi.org/10.1016/j.bjid.2012.12.010

41. EFMoH EFM of H. National Strategic Plan Tuberculosis and Leprosy Control 2006 – 2013 EC. Vol. 13. 2016.

42. Asres A, Jerene D, Deressa W. Tuberculosis treatment outcomes of six and eight month treatment regimens in districts of Southwestern Ethiopia : a comparative cross-sectional study. BMC Infect Dis [Internet]. 2016;1–8. Available from:

http://dx.doi.org/10.1186/s12879-016-1917-0

43. Legesse T, Admenur G, Gebregzabher S, Woldegebriel E, Fantahun B, Tsegay Y, et al. Tuberculosis (TB) in the refugee camps in Ethiopia: trends of case notification, profile, and treatment outcomes, 2014 to 2017. BMC Infect Dis. 2021 Dec 1;21(1).

44. WHO (World Health Organization). Global Tuberculosis Report 2019. 2019.

45. WHO (World Health Organization). The end TB strategy. 2015;

46. Worku S, Derbie A, Mekonnen D, Biadglegne F. Treatment outcomes of tuberculosis patients under directly observed treatment short-course at Debre Tabor General Hospital , northwest Ethiopia : nine-years retrospective study. 2018;1–7.

47. Habteyes Hailu Tola, Azar Tol, Davoud Shojaeizadeh GG. Tuberculosis Treatment Non-Adherence and Lost to Follow Up among TB Patients with or without HIV in Developing Countries: A Systematic Review. Iran J Public Health [Internet]. 2015 [cited 2021 Sep 24];44(1):1. Available from: /pmc/articles/PMC4449995/

48. Padmapriyadarsini, Narendran G, Swaminathan S. Diagnosis & treatment of tuberculosis in HIV co-infected patients. Indian J Med Res [Internet]. 2011 [cited 2021 Sep 24];134(6):850. Available from: https://www.ijmr.org.in/article.asp?issn=0971-5916;year=2011;volume=134;issue=6;spage=850;epage=865;aulast=Padmapriyadarsini

49. Addis Ababa Central Statistical Authority. Statistical Report for Addis Ababa City Administration. Vol. 3. 2007.

50. Gezahegn Berhe A, Erena B, Hassen M, Mamaru TL, Soressa YA. CITY PROFILE ADDIS ABABA SES Social Inclusion and Energy Managment for Informal Urban Settlements. 2017 [cited 2021 Sep 24]; Available from: www.eiabc.edu.et

Click here to access/download
Supporting Information
Desalegn_rawdata.xlsx

Impact of GeneXpert on TB diagnosis and anti-TB treatment outcome at health facilities in Addis Ababa, Ethiopia: In the post-MDG years

Desalegn Addise[1,2*], Laura E. Layland[2], Achim Hoerauf[2,3&], Biniam Wondale[4]

[1] Ethiopian Public Health Institute, Addis Ababa, Ethiopia

[2] Institute of Medical Microbiology, Immunology and Parasitology, University Hospital Bonn, Bonn, Germany

[3] German Center for Infection Research (DZIF), partner site Bonn-Cologne, Germany

[4] Department of Biology, Arba Minch University, Arba Minch, Ethiopia

*Corresponding author: Email: desalegnaddise@gmail.com

Abstract

Background: GeneXpert is an effective and rapid molecular system used for tuberculosis (TB) diagnosis. It is expected to improve the detection rate and treatment outcome to meet the sustainable development goal (SDG) and End TB strategy target set for 2030. This study aimed to evaluate the impact of GeneXpert on diagnosis and anti-TB treatment outcomes in the post-millennium development goals (MDGs) in the capital city of Ethiopia. Hence, the global priority indicator based on the End TB Strategy for TB treatment success rate was met early in 2018 in~~at~~ Addis Ababa, Ethiopia, which is anticipated to be met by 2025.

Method: A retrospective health facilities-based study was conducted in Addis Ababa, Ethiopia. Records of all TB cases diagnosed and treated in selected health facilities from January 2015 to December 2018 were reviewed and included in the study. Data analysis of descriptive and inferential statistics was conducted using SPSS version 20.

Result: The reviewed records have shown~~ed~~ that a total of 45,158 presumptive pulmonary TB (PTB) cases had accessed TB diagnosis services. Of which, 28.9% (13072/45158) were tested by AFB microscopy and 71.1% (32086/45158) were tested by GeneXpert. In the study period, the coverage of Xpert MTB/RIF testing was increased to 94.9% in 2018 compared to 1.6% in 2015. The number of presumptive PTB cases, who tested with the GeneXpert system compared to smear microscopy showed a significant increase. The odds of positivity were detected in males compared to females. The odds of detecting TB cases were~~as~~ much higher among study participants aged 15-44 years compared to younger than 15 years. Treatment success rate showed a relative improvement each year between 2015 and 2018 with a mean of 92.6%. Reduced odds of treatment successes were observed in age categories older than 35 years, and in TB/HIV co-infected patients. Increased odds of treatment successes were reported in the years between 2016 and 2018 compared to 2015.

Conclusion: Scaling up the Xpert MTB/RIF assay as a point-~~-~~of-~~-~~care test for presumptive TB cases in ~~the~~ resource-limited settings would have a significant impact to meet the SDG and End TB strategy both in~~ the improvement of~~ TB detection ~~rate~~ and ~~positive~~ treatment success rates.

Keywords: Tuberculosis, Treatment outcomes, GeneXpert, MDG, SDG, Addis Ababa, Ethiopia

Background

Tuberculosis (TB) is a chronic infectious disease mainly caused by *Mycobacterium tuberculosis* that affects primarily the lung. ~~According to the 2020 global TB report, t~~There were an estimated 10 million TB cases. ~~I~~In 2019. Of which, ~~a total of~~ there were 206,030 multi-~~-~~drug resistant (MDR) including rifampicin resistance (RR), and 12,350 laboratory-confirmed extensively drug resistance (XDR) TB cases~~ were reported~~. ~~In the same year, a~~About 1.4 million deaths ~~were~~ happened due to TB [1].

In sub-Saharan African countries, the rapid increase in~~ emerging~~ drug-resistance TB is a challenge [2]. ~~For this, t~~The Xpert MTB/RIF assay is a solution to detect *M. tuberculosis* including RR cases at the same time [3,4]. ~~Early diagnosis of TB cases has at most importance for the successful treatment outcomes.~~ In sputum samples with negative smear results, the Xpert MTB/RIF assay has a pooled sensitivity of 60.6-77% and ~~a pooled~~ specificity of 98.8% [5]. Due to its improved sensitivity and specificity ~~of Xpert MTB/RIF assay~~ over smear microscopy for the diagnosis of TB, the Xpert MTB/RIF assay i~~wa~~s~~were~~ promised to improve the anti-TB treatment outcome [6–9]. ~~Early diagnosis of TB cases has at most importance for successful treatment outcomes.~~ Xpert MTB/RIF assay reduces the anti-TB treatment initiation time by around four weeks for patients with paucibacillary for *M. tuberculosis* [6,10]. With these conditions, World Health Organization (WHO) endorsed and recommended the use of the GeneXpert system at the point of care for the diagnosis of TB [11].

Proper diagnosis and treatment of TB are~~is~~ crucial for the effort towards meeting the sustainable development goal (SDG) and End TB strategy targets set for 2035. Ethiopia ~~had~~ achieved the TB-~~-~~related Millennium Development Goals (MDGs) with a~~the~~ reduction of deaths due to TB by 75% and the TB incidence rate by more than 50% in 2015 compared to 1990 [2]. The GeneXpert instruments were introduced and have been in use in Ethiopia since 2012 [12]. They are used to diagnose only selected presumptive TB cases (presumptive DR-TB, HIV positive, children, and extrapulmonary TB), while the remaining cases with low-risk for DR-TB, HIV negative, and adult are diagnosed by sputum-smear microscopy [13]. Sputum-smear microscopy is a first-line test despite having a very poor sensitivity (25-75% compared to culture) and being unable to detect drug-resistant strains [14]. Since 2017, the

GeneXpert system is in use to diagnose all presumptive TB cases presented with signs and symptoms of~~for~~ TB at any given health facility in Addis Ababa. However, the impact of Xpert MTB/RIF assay in TB diagnosis and treatment outcome in health facilities of Addis Ababa is not evaluated. Therefore, ~~the aim of~~ this study ~~was~~ aimed to review the available records at the selected health facilities in Addis Ababa, Ethiopia, and to evaluate the impacts of Xpert MTB/RIF assay on the number of TB cases detected and successes in anti-TB treatment outcome~~s~~.

Methods

Study setting

The study was conducted in 19 public health facilities, which provide TB diagnosis services by the Xpert MTB/RIF assay in Addis Ababa, Ethiopia. Addis Ababa is the largest and capital city covering an area of 540 km^2 with an estimated population of 3,384,569 according to the 2007 census (World population review 2020). The city administration is divided into 10 sub-cities.

The government health infrastructure of Addis Ababa includes hospitals, health centers, and Addis Ababa Public Health Research and Emergency Management (AAPHREM) hereafter we call Addis Ababa Regional Reference Laboratory (A.A. Ref. Lab).

Study design, data collection, and ethical review

A retrospective health facility-based study was conducted in the capital city of Ethiopia by reviewing the laboratory TB registry log~~-~~book and TB unit register. In Addis Ababa, the Xpert MTB/RIF testing service is provided in five hospitals, 18 health centers, and one regional reference laboratory. The type of methods used for TB diagnosis and the number~~s~~ of presumptive pulmonary TB cases tested in the study period between January 2015 and December 2018 were recorded from the laboratory TB registry log~~-~~book. All tests for all age groups of presumptive pulmonary TB cases were done using sputa as a sample source. All TB cases on anti-TB treatment, which were registered in the respective health facilities' TB unit register during the study period, were included in the study. Out of 24 health facilities, which provided Xpert MTB/RIF testing services, five health facilities were excluded from the study.

Of the five excluded health facilities, four health facilities did not document TB registry log books and one health facility refused to participate in the study. Additional two health facilities were excluded from treatment outcome analysis since they only provided test service but not DOTs service. Hence, the laboratory data were~~as~~ collected from 19 health facilities whereas anti-TB treatment outcome data were~~as~~ collected from 17 health facilities.

Demographic and clinical data of TB cases were reviewed and recorded into Microsoft Excel by a data ~~-~~clerk. The correctness of the recorded data was cross-checked by the principal investigator.

Ethical clearance was obtained from the Ethical Review Committee of the University Hospital Bonn~~,~~ and the Addis Ababa Regional Reference Laboratory. In addition, a support letter was obtained from the University Hospital Bonn, Institute of Hygiene and Public Health. The selected health facilities were informed about the study and written consent was obtained. The collected data privacy and confidentiality were protected at all times during the data collection period. All participants' identifiers were removed during analysis once the data collection and quality assurance processes were completed.

Operational definitions:

The following treatment outcomes are defined as follows per WHO guideline 2013.

Cured – A pulmonary TB patient with bacteriologically confirmed TB at the beginning of treatment who was smear- or culture-negative in the last month of treatment and on at least one previous occasion.

Treatment completed – A TB patient who completed treatment without evidence of failure BUT

with no record to show that sputum smear or culture results in the last month of treatment and on at least one previous occasion were negative, either because tests were not done or because results are unavailable.

Treatment failed – A TB patient whose sputum smear or culture is positive at month 5 or later during treatment.

~~during treatment.~~

Formatted: Font: 12 pt

Formatted: Justified, Space After: 0 pt, Line spacing: 1.5 lines

Formatted: Justified, Space After: 0 pt

Formatted: Font: 12 pt

Formatted: Justified, Space After: 0 pt, Line spacing: 1.5 lines

Formatted: Font: 12 pt

Formatted: Justified, Space After: 0 pt, Line spacing: 1.5 lines

Formatted: Font: 12 pt

Died – A TB patient who dies for any reason before starting or during the course of treatment.

Loss to follow-up – A TB patient who did not start treatment or whose treatment was interrupted for two~~2~~ consecutive months or more.

Not evaluated - A TB patient for whom no treatment outcome is assigned. This includes cases "transferred out" to another treatment unit as well as cases for whom the treatment outcome is unknown to the reporting unit.

Transfer-in - patients have been transferred from another TB register to continue treatment.

Successful treatment outcome – the sum of cured and treatment completed.

Formatted: Font: 12 pt

Formatted: Justified, Space After: 0 pt, Line spacing: 1.5 lines

Formatted: Justified, Space After: 0 pt

Formatted: Font: 12 pt

Data analysis and statistics

The data ~~in~~ were primarly recorded in Microsoft Excel ~~were~~ and then exported into SPSS Version 20.0 software (SPSS INC, Chicago, IL, USA) for analysis. The findings were presented using descriptive statistics. Bivariate and multivariate logistic regression models were also used to evaluate the association of independent variables with dependent variables. The level of significance was set at $P \leq 0.05$.

Results

Trends of presumptive pulmonary TB cases diagnosis from smear microscopy to GeneXpert system

The reviewed records have shown~~ed~~ that a total of 45,158 presumptive pulmonary TB cases had accessed TB diagnosis services. Of the total presumptive pulmonary TB cases in the study period, 28.9% (13072/45158) were tested by acid-~~-~~fast bacilli (AFB) microscopy ~~and~~ whereas 71.1% (32086/45158) were tested by GeneXpert system Table 1.~~-~~ Xpert MTB/RIF testing coverage increased during the study period from 1.6% in 2015 to 94.9% in 2018, despite a reduction in AFB microscopy utilization from 98.4% in 2015 to 5.1% in 2018~~In the study period, the coverage of Xpert MTB/RIF testing was increased from 1.6% in 2015 to 94.9% in 2018 while the AFB microscopy usage was decreased from 98.4% in 2015 to 5.1% in 2018~~.

Table 1. Uptake of GeneXpert system compared to smear microscopy for TB diagnosis at Addis Ababa, Ethiopia, between 2015 and 2018.

Year	Method of Diagnosis		Total N (%)
	Microscopy N (%)	GeneXpert system N (%)	
2015	4560 (98.4)	75 (1.6)	4635 (10.3)
2016	5679 (61.2)	3597 (38.8)	9276 (20.5)
2017	1931 (14.1)	11744 (85.9)	13675 (30.3)
2018	902 (5.1)	16670 (94.9)	17572 (38.9)
Total	13072 (28.9)	32086 (71.1)	45158 (100.0)

TB positivity and associated factors within presumptive TB cases

After adjusting factors associated with TB case detection, the GeneXpert system had~~ a~~ higher odds of detecting TB cases (AOR: 4.78, 95 % CI: 4.13 – 5.53) compared to smear microscopy. Presumptive TB cases were more likely to be diagnosed with TB if they were male (AOR: 1.45, 95% CI: 1.36–1.54) compared to female cases~~s~~. The odds of detecting TB cases increased among study participants aged 15-44 years compared to study participants younger than 15 years Table 2.

Table 2. Factors associated with the positive results of presumptive TB cases in~~at~~ Addis Ababa, Ethiopia between 2015 and 2018

Variable	Total N (%)	Positive case N (%)	**COR (95% CI)**	**AOR (95% CI)**	**P-value**
Sex[a]					
F	22882 (50.7)	2081 (9.1)	1	1	
M	22242 (49.3)	2694 (12.1)	**1.38 (1.30 – 1.46)**	**1.45 (1.36 – 1.54)**	<0.001
Age[b]					
0-14	2309 (5.1)	113 (4.9)	1	1	
15-24	8359 (18.6)	1273 (15.2)	**3.49 (2.86 – 4.26)**	**3.72 (3.05 – 4.55)**	<0.001
25-34	11769 (26.2)	1582 (13.4)	**3.02 (2.48 – 3.67)**	**3.03 (2.48 – 3.69)**	<0.001
35-44	8075 (18.0)	822 (10.2)	**2.20 (1.80 – 2.70)**	**2.08 (1.69 – 2.55)**	<0.001
>=45	14436 (32.1)	809 (5.6)	1.15 (0.94 – 1.41)	1.11 (0.91 – 1.36)	0.313
Methods of Dx					
Microscope	13072 (28.9)	846 (6.5)	1	1	
GeneXpert	32086 (71.1)	3963 (12.4)	**2.04 (1.89 – 2.20)**	**4.78 (4.13 – 5.53)**	<0.001
Year of Dx					
2015	4635 (10.3)	455 (9.8)	1	1	
2016	9276 (20.5)	1167 (12.6)	**1.32 (1.18 – 1.48)**	**0.72 (0.62 – 0.83)**	<0.001
2017	13675 (30.3)	1525 (11.2)	**1.15 (1.03 – 1.29)**	**0.37 (0.31 – 0.44)**	<0.001
2018	17572 (38.9)	1662 (9.5)	0.96 (0.86 – 1.07)	**0.28 (0.24 – 0.34)**	<0.001
Sub-cities					
Addis Ketema	3564 (7.9)	284 (8.0)	1	1	
Kolfe Keranyo	8101 (17.9)	744 (9.2)	**1.17 (1.01 – 1.35)**	**1.53 (1.31 – 1.77)**	<0.001
Bole	1645 (3.6)	194 (11.8)	**1.54 (1.27 – 1.87)**	**1.43 (1.17 – 1.75)**	<0.001
Akaki-Kality	4759 (10.5)	524 (11.0)	**1.43 (1.23 – 1.66)**	**1.59 (1.36 – 1.86)**	<0.001
Yeka	7980 (17.7)	683 (8.6)	1.08 (0.94 – 1.25)	**1.23 (1.06 – 1.44)**	0.006
Arada	4897 (10.8)	470 (9.6)	**1.23 (1.05 – 1.43)**	**1.41 (1.20 – 1.65)**	<0.001
Nifas Silk	7028 (15.6)	710 (10.1)	**1.30 (1.12 – 1.50)**	**1.48 (1.28 – 1.72)**	<0.001
Kirkos	1704 (3.8)	147 (8.6)	1.09 (0.89 – 1.34)	**1.32 (1.06 – 1.64)**	0.013
A.A. Ref. Lab*	5480 (12.1)	1053 (19.2)	**2.75 (2.39 – 3.16)**	**1.42 (1.20 – 1.69)**	<0.001

[a]- of the total 34 participants had no sex information; [b]- of the total 210 participants had no age information; * stands for Addis Ababa Regional Reference Laboratory.

Demographics and clinical characteristics of study participants on anti-TB treatment

The total numbers of patients who received anti-~~-~~TB treatment in 17 health facilities ~~with GeneXpert system~~ were 8446 including extra~~-~~pulmonary TB (EPTB) cases. Table 3 details the d~~D~~emographic and clinical characteristics of TB patients who received anti-TB treatment during the study periods~~ were described in Table 3~~.

The majority, 77% (6504/8446) of TB patients were in the age group 15-44 years. Male patients 51.7% (4365/8446), who received treatment in the study period were slightly higher than females. The percentages of patients with no anti-TB treatment history, re-treatment, and transfer-in cases were 86.3% (7282/8446), 9.4% (797/8446), and 4.3% (367/8446), respectively. TB/HIV co-infection was 20.4% (1723/8446). TB cases in the study period were PTBN 26.7% (2252/8446), PTBP 29.6% (2500/8446), and EPTB 43.7% (3694/8446).

Table 3. Demographics and clinical characteristics of TB patients who received anti-TB treatment in Addis Ababa, Ethiopia between 2015 and 2018.

**Variables**		**TB Types in Number (%)**			**Total N (%)**
		**PTBN**	**PTBP**	**EPTB**	
Sex					
	F	992 (44.0)	1097 (43.9)	1992 (53.9)	4081 (48.3)
	M	1260 (56.0)	1403 (56.1)	1702 (46.1)	4365 (51.7)
Age					
	< 15	84 (3.7)	53 (2.1)	178 (4.8)	315 (3.7)
	15-24	533 (23.7)	763 (30.5)	1012 (27.4)	2308 (27.3)
	25-34	596 (26.5)	895 (35.8)	1213 (32.9)	2704 (32.0)
	35-44	431 (19.1)	418 (16.7)	643 (17.4)	1492 (17.7)
	45-54	257 (11.4)	202 (8.1)	283 (7.7)	742 (8.8)
	>=55	351 (15.6)	169 (6.8)	359 (9.7)	879 (10.4)
	Unknown	0 (0.0)	0 (0.0)	6 (0.1)	6 (0.1)
TB History Category					
	New	1871 (83.1)	2134 (85.4)	3277 (88.7)	7282 (86.3)
	Re-treatment	282 (12.5)	238 (9.5)	277 (7.5)	797 (9.4)
	Transfer in	99 (4.4)	128 (5.1)	140 (3.8)	367 (4.3)
HIV Status of TB Cases					
	Non-infected	1627 (72.2)	1920 (76.8)	2812 (76.1)	6359 (75.3)
	Infected	532 (23.6)	476 (19.0)	715 (19.4)	1723 (20.4)
	Unknown	93 (4.1)	104 (4.2)	167 (4.5)	364 (4.3)
Year of Registration					
	2015	726 (32.2)	585 (23.4)	976 (26.4)	2287 (27.1)
	2016	668 (29.7)	536 (21.4)	946 (25.6)	2150 (25.5)
	2017	458 (20.3)	636 (25.4)	874 (23.7)	1968 (23.3)
	2018	400 (17.8)	743 (29.7)	898 (24.3)	2041 (24.2)
Sub-cities in Addis Ababa					
	Addis Ketema	147 (6.5)	144 (5.8)	236 (6.4)	527 (6.2)
	Kolfe Keranyo	610 (27.1)	720 (28.8)	1003 (27.2)	2333 (27.6)
	Bole	122 (5.4)	148 (5.9)	205 (5.5)	475 (5.6)
	Akaki-Kaliti	176 (7.8)	196 (7.8)	229 (6.2)	601 (7.1)
	Yeka	606 (26.9)	506 (20.2)	753 (20.4)	1865 (22.1)
	Arada	27 (1.2)	37 (1.5)	83 (2.2)	147 (1.7)

Nefas Silk	518 (23.0)	701 (28.0)	984 (26.6)	2203 (26.1)
Kirkos	46 (2.0)	48 (1.9)	201 (5.4)	295 (3.5)
**Total**	**2252 (26.7)**	**2500 (29.6)**	**3694 (43.7)**	**8446 (100)**

PTBN - Pulmonary TB clinically; **PTBP** - Pulmonary TB bacteriologically; **EPTB** - Extra-pulmonary TB

Treatment outcome

O~~In o~~verall, 7478 out of 8080 TB patients (92.6%)~~92.6% (7478/8080) of TB patients~~ had~~ve~~ successful treatment outcomes. Of these, 27.3% (2205/8080) patients had been~~were~~ cured and 65.3% (5273/8080) patients ~~were~~ had completed treatment~~ completed~~. ~~Less treatment success was recorded in~~ HIV~~-~~-infected TB patients had a lower rate of treatment success 86.8% (1416/1632) compared to non-infected ones 94.2% (5765/6121). Treatment outcomes after 2015 showed relative improvement ~~after the year 2015~~ Table 4.

Table 4. Treatment outcomes of TB patients at Addis Ababa, Ethiopia between 2015 and 2018.

**Variables**	**Treatment Outcome[a] in Number (%)**					**Total N (%)**	**P-value**
	**Successful**		**Unsuccessful**				
	**Cured**	**T. Completed**	**Died**	**T. Failed**	**Loss to Follow-Up**		
Sex							
F	965 (24.7)	2676 (68.6)	22 (0.6)	196 (5.0)	44 (1.1)	3903 (48.3)	< 0.001
M	1240 (29.7)	2597 (62.2)	26 (0.6)	222 (5.3)	92 (2.2)	4177 (51.7)	
Age Category in years							
< 15	48 (15.5)	249 (80.6)	1 (0.3)	8 (2.6)	3 (1.0)	309 (3.8)	< 0.001
15-24	689 (30.9)	1439 (64.5)	20 (0.9)	44 (2.0)	38 (1.7)	2230 (27.6)	
25-34	789 (30.5)	1662 (64.3)	11 (0.4)	86 (3.3)	37 (1.4)	2585 (32.0)	
35-44	366 (25.7)	922 (64.7)	9 (0.6)	104 (7.3)	24 (1.7)	1425 (17.6)	
45-54	175 (24.9)	444 (63.2)	2 (0.3)	66 (9.4)	15 (2.1)	702 (8.7)	
>=55	138 (16.8)	552 (67.1)	5 (0.6)	110 (13.4)	18 (2.2)	823 (10.2)	
Unknown	0 (0.0)	5 (83.3)	0 (0.0)	0 (0.0)	1 (16.7)	6 (0.1)	
TB History Category							
New	1916 (27.3)	4604 (65.6)	45 (0.6)	344 (4.9)	109 (1.6)	7018 (86.9)	0.005
Re-treatment	189 (25.9)	468 (64.0)	2 (0.3)	56 (7.7)	16 (2.2)	731 (9.0)	
Transfer in	100 (30.2)	201 (60.7)	1 (0.3)	18 (5.4)	11 (3.3)	331 (4.1)	
TB Type							
PTBN	NA	1994 (91.6)	7 (0.3)	140 (6.4)	35 (1.6)	2176 (26.9)	< 0.001
PTBP	2205 (92.9)	0 (0.0)	29 (1.2)	82 (3.5)	58 (2.4)	2374 (29.4)	

EPTB	NA	3279 (92.9)	12 (0.3)	196 (5.6)	43 (1.2)	3530 (43.7)	
HIV Status of TB Cases							
Non-infected	1738 (28.4)	4027 (65.8)	38 (0.6)	211 (3.4)	107 (1.7)	6121 (75.8)	< 0.001
Infected	384 (23.5)	1032 (63.2)	9 (0.6)	184 (11.3)	23 (1.4)	1632 (20.2)	
Unknown	83 (25.4)	214 (65.4)	1 (0.3)	23 (7.0)	6 (1.8)	327 (4.0)	
Year of Registration							
2015	488 (22.8)	1452 (67.9)	17 (0.8)	134 (6.3)	49 (2.3)	2140 (26.5)	<0.001
2016	469 (22.7)	1453 (70.3)	11 (0.5)	100 (4.8)	35 (1.7)	2068 (25.6)	
2017	575 (30.0)	1215 (63.4)	8 (0.4)	89 (4.6)	29 (1.5)	1916 (23.7)	
2018	673 (34.4)	1153 (58.9)	12 (0.6)	95 (4.9)	23 (1.2)	1956 (24.2)	
Sub-cities							
Addis Ketema	119 (23.8)	337 (67.3)	5 (1.0)	29 (5.8)	11 (2.2)	501 (6.2)	<0.001
Kolfe Keranyo	636 (28.2)	1459 (64.7)	9 (0.4)	119 (5.3)	31 (1.4)	2254 (27.9)	
Bole	141 (30.5)	298 (64.4)	2 (0.4)	22 (4.8)	0 (0.0)	463 (5.7)	
Akaki-Kaliti	175 (29.6)	369 (62.4)	8 (1.4)	29 (4.9)	10 (1.7)	591 (7.3)	
Yeka	451 (25.3)	1195 (66.9)	11 (0.6)	99 (5.5)	30 (1.7)	1786 (22.1)	
Arada	34 (24.8)	91 (66.4)	0 (0.0)	12 (8.8)	0 (0.0)	137 (1.7)	
Nefas Silk	612 (29.1)	1341 (63.8)	13 (0.6)	88 (4.2)	47 (2.2)	2101 (26.0)	
Kirkos	37 (15.0)	183 (74.1)	0 (0.0)	20 (8.1)	7 (2.8)	247 (3.1)	
**Total**	**2205 (27.3)**	**5273 (65.3)**	**48 (0.6)**	**418 (5.2)**	**136 (1.7)**	**8080 (100.0)**	

[a] TB patients whose treatment outcome is not evaluated/recorded (301) and RR-TB cases transferred to MDR Treatment initiating centers (65) were excluded during analysis. NA- Not applicable; T. completed- treatment completed; T. failed- treatment failed

Treatment success and associated factors

After adjusting factors associated with treatment outcome, age categories 35-44 (AOR: 0.49, 95% CI: 0.27 – 0.90), 45-54 (AOR: 0.36, 95% CI: 0.19 – 0.67), > =55 (AOR: 0.20, 95% CI: 0.11 – 0.37) and TB/HIV co-infected patients (AOR: 0.44 95% CI: 0.36 – 0.53) were predicted with lower odds of successful treatment outcome. Whereas, patients receiving treatment at Amoraw Health Center in Bole Sub-city had a better treatment success rate (AOR: 1.73, 95% CI: 1.03 – 2.91) compared to Addis Ketema Health Center in Addis Ketema Sub-city Table 5.

Table 5. Factors associated with successful treatment outcomes of TB patients in~~at~~ Addis Ababa, Ethiopia between 2015 and 2018.

Variables	Total N (%)	Treatment success N (%)	COR (95% CI)	AOR (95% CI)	p-value
Sex					
F	3903 (48.3)	3641 (93.3)	1	1	
M	4177 (51.7)	3837 (91.9)	**0.81 (0.69 – 0.96)**	0.90 (0.76 – 1.08)	0.268
Age					
0-14	309 (3.8)	297 (96.1)	1	1	
15-24	2230 (27.6)	2128 (95.4)	0.84 (0.46 – 1.55)	0.76 (0.41 – 1.40)	0.378
25-34	2585 (32.0)	2451 (94.8)	0.74 (0.40 – 1.35)	0.77 (0.42 – 1.42)	0.407
35-44	1425 (17.6)	1288 (90.4)	**0.38 (0.21 – 0.69)**	**0.49 (0.27 – 0.90)**	**0.021**
45-54	702 (8.7)	619 (88.2)	**0.30 (0.16 – 0.56)**	**0.36 (0.19 – 0.67)**	**0.001**
>=55	823 (10.2)	690 (83.8)	**0.21 (0.11 – 0.38)**	**0.20 (0.11 – 0.37)**	**<0.001**
Unknown	6 (0.1)	5 (83.3)	0.20 (0.02 – 1.87)	0.23 (0.02 – 2.20)	0.201
TB History Category					
New	7018 (86.9)	6520 (92.9)	1	1	
Re-treatment	731 (9.0)	657 (89.9)	**0.68 (0.52 – 0.88)**	0.90 (0.69 – 1.18)	0.451
Transfer in	331 (4.1)	301 (90.9)	0.77 (0.52 – 1.13)	0.78 (0.53 – 1.17)	0.229
TB Type					
PTBN	2176 (26.9)	1994 (91.6)	1	1	
PTBP	2374 (29.4)	2205 (92.9)	1.19 (0.96 – 1.48)	0.93 (0.74 – 1.16)	0.504
EPTB	3530 (43.7)	3279 (92.9)	1.19 (0.98 – 1.46)	0.98 (0.80 – 1.21)	0.863
HIV Status of TB Cases					
Non-infected	6121 (75.8)	5765 (94.2)	1	1	
Infected	1632 (20.2)	1416 (86.8)	**0.41 (0.34 – 0.48)**	**0.44 (0.36 – 0.53)**	**<0.001**
Unknown	327 (4.0)	297 (90.8)	**0.61 (0.41 – 0.90)**	**0.60 (0.40 – 0.91)**	**0.016**
Year of Registration					
2015	2140 (26.5)	1940 (90.7)	1	1	
2016	2068 (25.6)	1922 (92.9)	**1.36 (1.09 – 1.70)**	**1.39 (1.10 – 1.75)**	**0.006**
2017	1916 (23.7)	1790 (93.4)	**1.47 (1.16 – 1.85)**	**1.42 (1.12 - 1.80)**	**0.004**

2018	1956 (24.2)	1826 (93.4)	**1.45 (1.15 – 1.82)**	**1.43 (1.13 – 1.81)**	**0.003**
Sub-cities					
Addis Ketema	501 (6.2)	456 (91.0)	1	1	
Kolfe Keranyo	2254 (27.9)	2095 (92.9)	1.30 (0.92 – 1.84)	1.30 (0.91 – 1.85)	0.148
Bole	463 (5.7)	439 (94.8)	**1.81 (1.08 – 3.01)**	**1.73 (1.03 – 2.91)**	**0.039**
Akaki-Kaliti	591 (7.3)	544 (92.0)	1.14 (0.75 – 1.75)	1.21 (0.78 – 1.88)	0.387
Yeka	1731 (21.4)	1595 (92.1)	1.16 (0.82 – 1.65)	1.21 (0.85 – 1.75)	0.289
Arada	192 (2.4)	176 (91.7)	1.09 (0.60 – 1.97)	1.38 (0.75 – 2.55)	0.299
Nefas Silk	2101 (26.0)	1953 (93.0)	1.30 (0.92 – 1.85)	1.28 (0.90 – 1.84)	0.171
Kirkos	247 (3.1)	220 (89.1)	0.80 (0.49 – 1.33)	1.13 (0.67 – 1.92)	0.650

Bold- values, which show significance (< 0.05); COR- crude odd ratio; AOR- adjusted odd ratio

4. Discussion

Early diagnosis of presumptive TB cases has great importance for improving the treatment outcome and reducing the risk of transmitting the disease to others. Xpert MTB/RIF assay has more sensitivity than smear microscopy in detecting TB cases [15–20]. This study was to evaluate the TB diagnosis and treatment outcome in health facilities of Addis Ababa, Ethiopia after the introduction of the GeneXpert system. The study showed that the trends of shifting pulmonary TB diagnosis methods from smear microscopy to GeneXpert system brought an improvement in case detection and success rate of treatment outcomes.

The introduction of Xpert MTB/RIF assay in Addis Ababa ~~had~~ increased the number of identified TB cases each year to almost four-fold in 2018 with reference to 2015. This might be due to the high sensitivity of Xpert MTB/RIF assay in *M. tuberculosis* detection [15–20]. This finding has been supported by the logistic regression analysis result of the current study. Presumptive TB cases ~~were~~ diagnosed with GeneXpert had 4.78 odds of being TB positive compared to those were diagnosed by microscopy. This finding was consistent with the previous studies performed on the advanced diagnostic performance of the GeneXpert system over smear microscopy that revealed a higher TB cases detection [17,21–26].

Male patients who were diagnosed with~~for~~ TB had higher odds (AOR: 1.45, 95% CI: 1.36–1.54) of being TB positive compared to females. This finding is concordant with~~to~~ findings from the 2016 Global Burden of Diseases and different studies conducted in different countries, particularly in low and middle-~~-~~income countries [27–33]. Such sex differences might attribute to biological TB susceptibility differences between males and females [34], but as indicated by Manish Gupta the underlying genetic mechanism is not clearly defined [35]. The economically productive age group, between 15 and 44 years, was 62.8% of patients diagnosed for TB and they had higher odds of being positive compared to the age group less than 14 years. This might be due to the travel history of the economically productive age group far from their home looking for education, jobs, and social interactions. These might increase the chance of being in contact with infected individuals. The other factor associated with children less than 14 years of age might be the sputum quality that is children are more unlikely to produce good-~~-~~quality sputum [36].

The male patients enrolled for DOTs service in the study period were 51.7%, which is comparable to reports from Addis Ababa 51% [37]. It is also found lower than findings from different parts of Ethiopia; 53.8% in Asella Teaching Hospital [38], 58% in Mekele [39], while ~~a~~ slightly higher than 46.8% in Addis Ababa [40]. Among the total TB cases that received anti-TB treatment 20.4% of TB cases were HIV-~~-~~positive. This was slightly higher than the HIV-positive rate reported in Northern Ethiopia 18.8% [39] but lower than among notified TB cases in 2016 in Addis Ababa 28% [41].

Among pulmonary TB-~~-~~positive cases, 92.9% were cured. Of all TB types, 92.6% of TB patients had successful treatment outcomes. The mean treatment success rate in this study was higher than treatment success~~es~~ rates reported in districts of South~~-~~western (88%), Asela Teaching Hospital (81.7%), and in the refugee camps (75.9%) in Ethiopia [38,42,43]. But relatively lower than the treatment outcome notified for new TB cases in 2017 in Ethiopia 96% [44]. This finding has revealed that a 30% decrease in death rate was seen in 2018 among patients on anti-TB treatment compared with 2015. The increase in trends of treatment success between consecutive years showed a relative improvement in each year between 2015 and 2018. The global priority indicator and target for all countries were planned to reach a 90% treatment success rate at the latest ~~by~~ 2025 [45]. In this regard, the End TB Strategy towards the TB treatment success rate was met early in 2018 in~~at~~ Addis Ababa, Ethiopia.

The odds of treatment outcome in age groups and TB/HIV co-infection in this study was in agreement with findings from Addis Ababa, Asella Teaching Hospital, and Debre Tabor General Hospital [37,38,46]. TB patients with ages >= 35 years and being co-infected with HIV were likely to have a lower treatment success rate. In elder age group, this might be due to non-adherence to TB treatment as a result of possible factors such as family responsibility and being busy with socio-economic activities [47]. In TB/HIV co-infected patients, it might be due to the presence of pills burden when both TB and ART drugs were taken which leads to drug-drug interactions, overlapping drug toxicities, and immune reconstitution syndrome [48]. Patients receiving anti-TB treatment at Amoraw HC in Bole Sub-city were predicted to have higher positive treatment outcomes compared to Addis Ketema HC in Addis Ketema Sub-city. This might be due to patients at Bole Sub-city having relatively good socio-economic and housing status compared to Addis Ketema Sub-city [49,50].

This study was able to evaluate the significance of diagnosing presumptive TB cases with GeneXpert system over microscopy by including 45,158 presumptive PTB cases and the TB treatment outcome by assessing records of 8080 TB cases in the study area. However, it has the following limitations: it did not include bacteriologically confirmed PTB cases which were diagnosed in two health facilities without TB treatment unit in the treatment outcome analysis~~, which were diagnosed in two health facilities with GeneXpert system without TB unit but expected to be treated in other health facilities~~; second due to the nature of retrospective secondary data, there were missing data like sex and age categories~~y~~. However, as we have included more health facilities and a large number of TB cases in the study, the effect of missing data was considered not to be significant; third this study did not assess other factors or actions taken in health facilities to improve treatment outcome, which might have a positive role in treatment outcome improvement in addition to GeneXpert system. Lastly, this study lacks comorbidities other than HIV such as diabetes, hypertension, obesity, cancer etc ~~wasere not considered in the data analysis~~ which might have an effect on TB treatment outcome~~s~~.

5. Conclusion

The introduction of the GeneXpert system in Addis Ababa, Ethiopia had increased the number of confirmed TB cases with odds of 4.78 compared to the smear microscopy. The success of treatment outcomes ~~was~~ steadily improved each year in the study period. The overall success rate was 92.6%. Therefore, scaling up the Xpert MTB/RIF assay as a point-~~-~~of-care test for presumptive TB cases in ~~the~~ resource-limited settings would have a significant impact o~~i~~n meeting the SDG and End TB strategy both in TB detection and anti-TB treatment outcome.

Authors' contributions

DA was the primary researcher, conceived the study, designed, participated in data collection, conducted data analysis, and drafted the manuscript for publication. LEL, AH, and BW participated in the study design, data analysis, result interpretation, and reviewed the initial and final drafts of the manuscript. All authors read and approved the final manuscript.

Competing interests

The authors declare that they have no competing interests.

Acknowledgments

The authors would like to thank the Institutes of Hygiene and Public Health and of Medical Microbiology, Immunology and Parasitology, University Hospital Bonn and Addis Ababa Public Health Research and Emergency Management Ethical Review Office for reviewing the study protocol and writing a support letter for health facilities. We would like to thank Tewodros Daniel, Ashenafi Alemu, and Alem Mekonnen for their unreserved cooperation throughout the course of data collection. We are also grateful to health professionals working at the study sites, because without their willingness, cooperation, and support it would not be possible to come up with these findings.

References

Commented [U1]: Number 13 should be replaced with FMoH 2014 https://www.researchgate.net/publication/363498981_Implementation_Guideline_for_GeneXpert_MTBRIF_Assay_in_Ethiopia/link/631ff4a8873eca0c0082a5fa/download

1. WHO (World Health Organization). Global Tuberculosis Report 2020. Geneva; 2020.
2. WHO (World Health Organization). Global Tuberculosis Report 2018. 2018.
3. Gesine Meyer-Rath, Lawrence Long, William Macleod, Ian Sanne, Kate Bistline, Wendy Stevens SR. The incremental cost of introducing Xpert® MTB/RIF into the South African national tuberculosis programme: Results of the National TB Cost Model 2011/12 - 2016/17. Johannesburg; 2016.
4. Raizada N, Sachdeva KS, Sreenivas A, Vadera B, Gupta RS, Parmar M, et al. Feasibility of Decentralised Deployment of Xpert MTB / RIF Test at Lower Level of Health System in India. 2014;9(2).
5. Rimal R, Shrestha D, Pyakurel S, Poudel R, Shrestha P, Rai KR, et al. Diagnostic performance of GeneXpert MTB/RIF in detecting MTB in smear-negative presumptive TB patients. BMC Infect Dis [Internet]. 2022 Dec 1 [cited 2023 Apr 7];22(1):1–7. Available from: https://bmcinfectdis.biomedcentral.com/articles/10.1186/s12879-022-07287-5
6. Kwak N, Choi SM, Lee J, Park YS, Lee C hoon, Lee S min, et al. Diagnostic Accuracy and Turnaround Time of the Xpert MTB / RIF Assay in Routine Clinical Practice. 2013;8(10):1–6.
7. Millman AJ, Dowdy DW, Miller CR, Brownell R, Metcalfe JZ, Cattamanchi A, et al. Rapid Molecular Testing for TB to Guide Respiratory Isolation in the U . S .: A Cost-Benefit Analysis. 2013;8(11):1–8.
8. Menzies NA, Cohen T, Lin H ho, Murray M, Salomon JA. Population Health Impact and Cost-Effectiveness of Tuberculosis Diagnosis with Xpert MTB / RIF : A Dynamic Simulation and Economic Evaluation. 2012;9(11).
9. Creswell J, Codlin AJ, Andre E, Micek MA, Bedru A, Carter EJ, et al. Results from early programmatic implementation of Xpert MTB / RIF testing in nine countries. 2014;

10. Omrani AS, Al-otaibi MF, Al-ateah SM, Al-onazi FM, Baig K, El-khizzi NA, et al. GeneXpert MTB / RIF Testing in the Management of Patients with Active Tuberculosis ; A Real Life Experience from Saudi Arabia. 2014;46(1):30–4.

11. WHO (World Health Organization), Programme GT. Automated Real-Time Nucleic Acid Amplification Technology for Rapid and Simultaneous Detection of Tuberculosis and Rifampicin Resistance: Xpert MTB/RIF Assay for the Diagnosis of Pulmonary and Extrapulmonary TB in Adults and Children: Policy update. World Heal Organ [Internet]. 2013 [cited 2020 Dec 26];1–79. Available from: www.who.int

12. Alemu A, Amare M, Ameni G, Kebede A, Beyene D, Yenew B, et al. Monitoring quality indicators for the Xpert MTB/RIF molecular assay in Ethiopia. PLoS One [Internet]. 2019 Nov 1 [cited 2023 Apr 6];14(11):e0225205. Available from: https://journals.plos.org/plosone/article?id=10.1371/journal.pone.0225205

13. FMoH Ethiopia. Implementation Guideline for GeneXpert MTB/RIF Assay in Ethiopia [Internet]. 20149 [cited 2023 Apr 6]. Available from: https://ephi.gov.et/wp-content/uploads/2014/04/Implementation-Guideline-for-GeneXpert-MTBRIF-Assay-in-Ethiopia-Nov-03-2....pdf

14. WHO, FMoH. Guidelines for Clinical and Programmatic Management of TB , TB / HIV , DR-TB and Leprosy in Ethiopia. 2021;7(August):1–249.

15. Maynard-Smith L, Larke N, Peters JA, Lawn SD. Diagnostic accuracy of the Xpert MTB/RIF assay for extrapulmonary and pulmonary tuberculosis when testing non-respiratory samples: A systematic review. BMC Infect Dis. 2014 Dec 31;14(1).

16. Sorsa A, Kaso M. Diagnostic performance of GeneXpert in tuberculosis–HIV co–infected patients at Asella Teaching and Referral Hospital, Southeastern Ethiopia: A cross sectional study. PLoS One [Internet]. 2021 Jan 1 [cited 2021 Sep 23];16(1). Available from: /pmc/articles/PMC7840185/

17. Habte D, Melese M, Hiruy N, Gashu Z, Jerene D, Moges F, et al. The additional yield of GeneXpert MTB/RIF test in the diagnosis of pulmonary tuberculosis among household contacts of smear positive TB cases. Int J Infect Dis [Internet]. 2016 Aug 1 [cited 2021 Mar 21];49:179–84. Available from: https://pubmed.ncbi.nlm.nih.gov/27401584/

18. Pinyopornpanish K, Chaiwarith R, Pantip C, Keawvichit R, Wongworapat K, Khamnoi P, et al. Comparison of Xpert MTB/RIF Assay and the Conventional Sputum Microscopy in Detecting Mycobacterium tuberculosis in Northern Thailand . Tuberc Res Treat. 2015;2015:1–6.

19. Steingart KR, Schiller I, Horne DJ, Pai M, Boehme CC, Dendukuri N. Xpert® MTB/RIF assay for pulmonary tuberculosis and rifampicin resistance in adults. Cochrane Database Syst Rev. 2014 Jan 21;2014(1).

20. Lange B, Khan P, Kalmambetova G, Al-Darraji HA, Alland D, Antonenka U, et al. Diagnostic accuracy of the Xpert® MTB/RIF cycle threshold level to predict smear positivity: A meta-analysis. Int J Tuberc Lung Dis. 2017 May 1;21(5):493–502.

21. Khadka P, Thapaliya J, Basnet RB, Ghimire GR, Amatya J, Rijal BP. Diagnosis of tuberculosis from smear-negative presumptive TB cases using Xpert MTB/Rif assay: A cross-sectional study from Nepal. BMC Infect Dis [Internet]. 2019 Dec 30 [cited 2021 Mar 21];19(1):1090. Available from: https://bmcinfectdis.biomedcentral.com/articles/10.1186/s12879-019-4728-2

22. Zahoor D, Farhana A, Kanth F, Bha M. Evaluation of smear microscopy and geneXpert for the rapid diagnosis of pulmonary and extrapulmonary tuberculosis in a tertiary care hospital in North India: a descriptive prospective study. Int J Res Med Sci [Internet]. 2018 Apr 25 [cited 2021 Mar 21];6(5):1756. Available from: www.msjonline.org

23. Neil M. Ampel M. Diagnosing TB with the Xpert Assay vs. AFB Smear Microscopy. NEJM J Watch [Internet]. 2019 Mar 25 [cited 2021 Mar 21];2019. Available from: https://www.jwatch.org/NA48799/2019/03/25/diagnosing-tb-with-xpert-assay-vs-afb-smear-microscopy

24. Orina GM, Ong'wen SA, Amolo S, Orindi OT, Oginga J. Comparative Study of Smear Microscopy, Gene Xpert and Culture and Sensitivity Assays in Detection of Mycobacterium tuberculosis on Sputum Samples among Tuberculosis Suspected Cases in Nyamira County Referral Hospital. 2017;

25. Taddese BD, Desalegn DM, Misganaw AS, Kitila KT, Hailu TK, Bika AT. Diagnostic

Performance of Xpert MTB/RIF Assay Versus Ziehl-Neelsen Method for the Diagnosis of Pulmonary Tuberculosis in Addis Ababa, Ethiopia. Open Microbiol J. 2018 Dec 13;12(1):390–6.

26. Arora D, Dhanashree B. Utility of smear microscopy and genexpert for the detection of mycobacterium tuberculosis in clinical samples. GERMS [Internet]. 2020 [cited 2021 Mar 4];10(2):81–7. Available from: /pmc/articles/PMC7330512/

27. Onifade DA, Bayer AM, Montoya R, Haro M, Alva J, Franco J, et al. Gender-related factors influencing tuberculosis control in shantytowns: A qualitative study. BMC Public Health [Internet]. 2010 Dec 29 [cited 2021 Mar 22];10(1):381. Available from: http://bmcpublichealth.biomedcentral.com/articles/10.1186/1471-2458-10-381

28. Diwan VK, Thorson A. Sex, gender, and tuberculosis [Internet]. Vol. 353, Lancet. Elsevier Limited; 1999 [cited 2021 Mar 22]. p. 1000–1. Available from: http://www.thelancet.com/article/S0140673699013185/fulltext

29. World Health Organization (WHO). Tuberculosis Gender in Research. 2005.

30. Yang W teng, Gounder CR, Akande T, Neve J walter De, Mcintire KN, Chandrasekhar A, et al. Barriers and Delays in Tuberculosis Diagnosis and Treatment Services : Does Gender Matter ? 2014;2014.

31. Thorson A, Hoa NP, Long NH, Allebeck P, Diwan VK. Do women with tuberculosis have a lower likelihood of getting diagnosed? Prevalence and case detection of sputum smear positive pulmonary TB, a population-based study from Vietnam. J Clin Epidemiol [Internet]. 2004 Apr [cited 2021 Mar 22];57(4):398–402. Available from: https://pubmed.ncbi.nlm.nih.gov/15135842/

32. Krishnan L, Akande T, Shankar A V, Mcintire KN, Gounder CR, Gupta A, et al. Gender-Related Barriers and Delays in Accessing Tuberculosis Diagnostic and Treatment Services : A Systematic Review of Qualitative Studies. 2014;2014.

33. Amare D, Deribe K, Dejene T, Tessema GA, Melaku YA, Lakew Y, et al. Tuberculosis Burden in Ethiopia from 1990 to 2016 : Evidence from the Global Burden of Diseases 2016 Study. 2018;

34. Neyrolles O, Quintana-Murci L. Sexual Inequality in Tuberculosis. PLOS Med [Internet]. 2009 Dec [cited 2022 Jun 16];6(12):e1000199. Available from: https://journals.plos.org/plosmedicine/article?id=10.1371/journal.pmed.1000199

35. Gupta M, Srikrishna G, Klein SL, Bishai WR. Genetic and hormonal mechanisms underlying sex-specific immune responses in tuberculosis. Trends Immunol. 2022 Aug 1;43(8):640–56.

36. Hare JD. Manual of Clinical Microbiology. Arch Pediatr Adolesc Med [Internet]. 1975 Jun 1 [cited 2021 Mar 20];129(6):754. Available from: http://archpedi.jamanetwork.com/article.aspx?doi=10.1001/archpedi.1975.02120430086036

37. Alem Y, Gebre-selassie S. Treatment Outcome of Tuberculosis Patients in Selected Health Centres in Addis Ababa : a Five Year Retrospective Study. 2017;1:5–12.

38. Tafess K, Tasew G, Mekit S, Beyen TK, Abera A, Solomon Sisay, et al. Treatment Outcomes of Tuberculosis at asella Teaching hospital , ethiopia : Ten Years ' retrospective aggregated Data. Front Med. 2018;5(February):1–9.

39. Abdulkader M, Aken I Van, Niguse S, Hailekiros H, Spigt M. Treatment outcomes and their trend among tuberculosis patients treated at peripheral health settings of Northern Ethiopia between 2009 and 2014 : a registry ‑ based retrospective analysis. BMC Res Notes [Internet]. 2019;10–4. Available from: https://doi.org/10.1186/s13104-019-4824-9

40. Getahun B, Ameni G, Medhin G, Biadgilign S. Original article Treatment outcome of tuberculosis patients under directly observed treatment in Addis Ababa , Ethiopia. Brazilian J Infect Dis [Internet]. 2013;17(5):521–8. Available from: http://dx.doi.org/10.1016/j.bjid.2012.12.010

41. EFMoH EFM of H. National Strategic Plan Tuberculosis and Leprosy Control 2006 – 2013 EC. Vol. 13. 2016.

42. Asres A, Jerene D, Deressa W. Tuberculosis treatment outcomes of six and eight month treatment regimens in districts of Southwestern Ethiopia : a comparative cross-sectional study. BMC Infect Dis [Internet]. 2016;1–8. Available from:

http://dx.doi.org/10.1186/s12879-016-1917-0

43. Legesse T, Admenur G, Gebregzabher S, Woldegebriel E, Fantahun B, Tsegay Y, et al. Tuberculosis (TB) in the refugee camps in Ethiopia: trends of case notification, profile, and treatment outcomes, 2014 to 2017. BMC Infect Dis. 2021 Dec 1;21(1).

44. WHO (World Health Organization). Global Tuberculosis Report 2019. 2019.

45. WHO (World Health Organization). The end TB strategy. 2015;

46. Worku S, Derbie A, Mekonnen D, Biadglegne F. Treatment outcomes of tuberculosis patients under directly observed treatment short-course at Debre Tabor General Hospital , northwest Ethiopia : nine-years retrospective study. 2018;1–7.

47. Habteyes Hailu Tola, Azar Tol, Davoud Shojaeizadeh GG. Tuberculosis Treatment Non-Adherence and Lost to Follow Up among TB Patients with or without HIV in Developing Countries: A Systematic Review. Iran J Public Health [Internet]. 2015 [cited 2021 Sep 24];44(1):1. Available from: /pmc/articles/PMC4449995/

48. Padmapriyadarsini, Narendran G, Swaminathan S. Diagnosis & treatment of tuberculosis in HIV co-infected patients. Indian J Med Res [Internet]. 2011 [cited 2021 Sep 24];134(6):850. Available from: https://www.ijmr.org.in/article.asp?issn=0971-5916;year=2011;volume=134;issue=6;spage=850;epage=865;aulast=Padmapriyadarsini

49. Addis Ababa Central Statistical Authority. Statistical Report for Addis Ababa City Administration. Vol. 3. 2007.

50. Gezahegn Berhe A, Erena B, Hassen M, Mamaru TL, Soressa YA. CITY PROFILE ADDIS ABABA SES Social Inclusion and Energy Managment for Informal Urban Settlements. 2017 [cited 2021 Sep 24]; Available from: www.eiabc.edu.et

Letter to the academic editor and reviewers

Subject: Submission of a revised manuscript

Title: Impact of GeneXpert on TB diagnosis and anti-TB treatment outcome at health facilities in Addis Ababa, Ethiopia: in the post-MDG years

Manuscript ID: PONE-D-21-40508R2

Dear academic editor and reviewer(s),

Thank you very much for your informative and constructive comments on the manuscript entitled "Impact of GeneXpert on TB Diagnosis and Anti-TB Treatment Outcome at Health Facilities in Addis Ababa, Ethiopia: In the Post MDG Years". We also appreciate for giving us the opportunity to submit the revised version of our manuscript. We addressed all the comments one by one as seen in the table below.

Comment	Author Response	Page Number
1. Can the author explain the discrepancy between the date of the implementation of GeneXpert (2017) and the data which include 2015 and 2016	Thank you for the comments.<br>We, the authors, would like to make clear the discrepancy related to the implementation of GeneXpert in Ethiopia;<br>• It is true that the GeneXpert instruments were introduced in Ethiopia and in use since 2012 (Alemu et al., 2019), to diagnose only selected presumptive TB cases (Presumptive DR-TB, HIV +ve, Children; and EPTB), while the remaining cases with low-risk to DR-TB, HIV-VE, and adult were diagnosed by AFB microscopy based on the algorithm (FMoH Ethiopia, 2014). But, since 2017,	

	GeneXpert system is in use to diagnose all presumptive TB cases presented with signs and symptoms of TB at any given health facility in Addis Ababa.<br>• It is because of selective TB diagnosis made by GeneXpert instruments were available in the years 2015 and 2016.	
2. Can the author confirm the methodology - here a flow diagram may be useful - outlining the sensitivity and specificity of microscopy as well as the exact staining and review process used	Thank you very much for the comments.<br>The sensitivity and specificity were not assessed since, in this investigation, we collected retrospective data from health facilities in Addis Ababa.<br>Both staining techniques (Ziel-Neelsen staining (ZN) or fluorescent auramine staining (LED FM)) were in use in Addis Ababa but the health facilities do not keep track of which staining techniques were employed; instead, the record indicates that the diagnosis was performed using sputum smear microscopy. Due to the lack of clarity in the registration books, we are unable to determine the exact staining techniques.	
3. This manuscript still requires extensive English language editing	Thank you for the comments and suggestions, and we made corrections per the comments.	
4. Reviewer #3:<br>• Data in Table 1 shows an increase in TB testing (Total), I would like to know the reasons for the increase uptake from 4 635 in year 2015 to 17 572 in Year 2018.<br>• Secondly, the method for diagnosing using smear, was it a single smear per participant? if so why do we see an increase in uptake as GeneXpert testing is introduced.	Thank you for the comments.<br>• It is true that since the GeneXpert instrument has been used for diagnosis of presumptive TB cases as a first-line test in the chosen health facilities in Addis Ababa, the number of presumptive TB cases diagnosed for TB has increased. The instrument's improved sensitivity and specificity, which increase the trust of clinicians working in the OPD, ease of use, the lack of a requirement for two sputum samples, and the suitability of the reagent/cartridge may all be contributing factors to the increased TB test.<br>• Based on the recommendation of the guideline for clinical and programmatic management of TB and TB/HIV in Ethiopia, two sputum samples (Spot-Spot) are required for AFB Microscopy, and one sputum smear positive result confirms TB diagnosis. In this study, we collected the	

	final AFB microscopy result of the patients from the laboratory registration book.	
5. Reviewer #4: - There are serious issues with style and grammar that affect comprehension and require editing<br><br>- There is inconsistency between information given in background and results: the background states that GXP was introduced in 2017, yet GXP data is presented from 2015 onwards<br><br>- The background lacks information on microscopy and GXP in terms of performance data/limit of detection<br><br>- The method section is flawed; there is no information on MTB testing algorithms in the laboratories; no information on which AFB microscopy method was used; no information on treatment regimens for MDR TB; extracted data and analysis thereof is not clearly stated<br><br>- The results section doesn't mention proportion of patients on DS or MDR treatment; patients with extra-pulmonary TB are included yet the methods section only mentions sputum samples-- how were these diagnosed and from which samples?; the impact of the GXP introduction on treatment outcomes is not mentioned in results	Thank you for the comments.<br><br>• Thank you for the comments and we made corrections per the comments.<br>• It is true that the GeneXpert instruments were introduced in Ethiopia and in use since 2012 (Alemu et al., 2019), to diagnose only selected presumptive TB cases (Presumptive DR-TB, HIV +ve, Children; and EPTB), while the remaining cases with low-risk to DR-TB, HIV-VE, and Adult are diagnosed by AFB microscopy based on the algorithm (FMoH Ethiopia, 2014). But, since 2017, GeneXpert system is in use to diagnose all presumptive TB cases presented with signs and symptoms of TB at any given health facility in Addis Ababa. It is because of selective TB diagnoses made by GeneXpert instruments were available in the years 2015 and 2016.<br>• The performance data is included on page 3 in the last paragraph (we added a sentence which describes the performance of AFB smear microscopy)<br>• Both staining techniques (Ziel-Neelsen staining (ZN) or fluorescent auramine staining (LED FM)) are in use in Addis Ababa, but the health facilities do not keep track of which staining techniques were employed; instead, the record indicates that the diagnosis was performed using sputum smear microscopy. Due to the lack of clarity in the laboratory registration books, we are unable to determine the exact staining techniques.<br>• 65 MDR patients who were moved to the MDR Treatment Initiating Center (TIC) for second-line therapy and are not included in this study, as noted under Table 4 on page 10. Therefore, we were unable to obtain their treatment records. As a result, MDR patients are excluded from the treatment	

and should be elaborated on; relevance of adding data for various 'sub-cities' not clear unless of local interest	outcomes analysis.<br>• Clinical diagnosis was the main method of diagnosis for extra-pulmonary TB patients. Based on the recorded data only 10 (0.3%) of the 3694 extra-pulmonary TB patients had laboratory diagnostic information, The data record simply states that the diagnosis was made using either smear microscopy or GeneXpert, the health facilities did not keep track of the sample types utilized. That is why we don't give due attention on the diagnosis of EPTB and we are unable to pinpoint the precise sample type utilized.<br>• The result section on page 10 and lines 10th and 11th discuss the impact of the GXP introduction on treatment results.<br>• We appreciate your concern and your suggestion, adding data for various 'sub-cities' will be pertinent to local interest.	
