# Supplementary material for: Impact of the use of GeneXpert on TB diagnosis and anti-TB treatment outcome at health facilities in Addis Ababa, Ethiopia in the post-millennium development years
Source: PLoS One. 2023 Aug 25;18(8):e0289917. doi: 10.1371/journal.pone.0289917 (PMC10456184; doi:10.1371/journal.pone.0289917)
Supplement: Attachment — Submitted filename: Response to Reviewers.doc [file pone.0289917.s002.doc]

**Letter to the academic editor and reviewers**

**Subject: Submission of a revised manuscript**

**Title: Impact of GeneXpert on TB diagnosis and anti-TB treatment outcome at health facilities in Addis Ababa, Ethiopia: in the post MDG years**

**Manuscript ID: PONE-D-21-40508**

Dear editor and reviewers,

Thank you very much for your informative and constructive comments on our manuscript entitled “Impact of GeneXpert on TB Diagnosis and Anti-TB Treatment Outcome at Health Facilities in Addis Ababa, Ethiopia: In the Post MDG Years”. We also appreciate you for giving us the opportunity to revise our manuscript and correct errors in the previous version. We addressed all the comments one by one as seen in the table below.

| **Editor Comment (Third response)** | **Author Response** | **Page Number** |
| --- | --- | --- |
| 1. Please note that funding information should not appear in the Acknowledgments section or other areas of your manuscript. We will only publish funding information present in the Funding Statement section of the online submission form. Please remove any funding-related text from the manuscript. | Thank you for the comment. Accordingly, all the funding information removed from the manuscript | 21 |
| **Editor Comment (Second response)** | **Author Response** | **Page Number** |
| 2. You may seek permission from the original copyright holder of Figure (1) to publish the content specifically under the CC BY 4.0 license. | Thank you for the comment. The figure removed | 5 |
| 3. Please state what role the funders took in the study.  If the funders had no role, please state: "The funders had no role in study design, data collection and analysis, decision to publish, or preparation of the manuscript." | Thank you for the suggestion. We incorporated the sentence accordingly. | NA |
| 4. Your ethics statement should only appear in the Methods section of your manuscript. | Thank you for the suggestion. Correction made accordingly. | 4&5 |
| 5. Please ensure that you refer to Tables 2 and 5 in your text as, if accepted, production will need this reference to link the reader to the Table. | Thank you for the suggestion. Correction made accordingly. | 6&10 |
| **Editor Comment (First response)** | **Author Response** | **Page Number** |
| 6. There needs to be extensive English language editing including ensuring terminology like the references to the GeneXpert are correct | Thank you for the comments and we made corrections per the comments. | All |
| 7. There was some concern that the treatment outcomes were the incorrect measure of utility. | We, the authors, would like to acknowledge your concern. As reported by Menzies et al. 2012 “Introduction of Xpert could substantially change TB morbidity and mortality through improved case-finding and treatment, with more limited impact on long-term transmission dynamics”. It is because of this that we used ‘the number of TB cases detected’ and ‘the positive treatment outcome’ as a measure of utility to see the impact of using GeneXpert MTB/RIF assay as a point of care testing. | NA |
| 8. The authors should also defined how they assess sensitivity and specificity performance | Thank you for your comment and suggestion. We do not assess the sensitivity and specificity of the performance rather we have evaluated the trends in the number of TB cases detected and the positive treatment outcomes in the study period. | NA |
|  |  |  |
| **Journal Requirements** | **Author Response** | **Page Number** |
| 1. Please ensure that your manuscript meets PLOS ONE's style requirements, including those for file naming. | Thank you for your comment and suggestion. We have made a change accordingly. | All |
| 2. We note that Figure (1) in your submission contain copyrighted images. All PLOS content is published under the Creative Commons Attribution License (CC BY 4.0), which means that the manuscript, images, and Supporting Information files will be freely available online, and any third party is permitted to access, download, copy, distribute, and use these materials in any way, even commercially, with proper attribution.  We require you to either (1) present written permission from the copyright holder to publish these figures specifically under the CC BY 4.0 license, or (2) remove the figures from your submission: | The figure removed | 5 |
| **Reviewer 1 Comments** | **Author Response** | **Page Number** |
| The manuscript doesn’t read well so I suggest scientific editing. Word usage is incorrect eg. In the abstract, GeneXpert is described as an effective molecular diagnostic test, yet the GeneXpert is an analyzer, not a test. | Thank you for the comments and we made corrections per the comments. | All |
| Some sentences are too long and difficult to follow eg. Sentence 2 in the Background section. | Thank you for the suggestion. We made a change per the suggestion. | 3 |
| 1a. Background: Second paragraph, Line 4: Not sure how “acceptable” and “excellent” descriptions for the sensitivity and specificity were assigned. Please clarify. | Thank you for the comments and we made corrections to the paragraph per the comments. | 3 |
| 1b. Background: Second paragraph, line 8: is the word “TB” supposed to follow the word “paucibacillary?” | Thank you for the suggestion. We made a correction per the suggestion. | 3 |
| 2. Study design and data collection Line 8: Five health centres were excluded due to refusal – not sure what this refers to. | Thank you for the suggestion. We made a correction per the suggestion.   - To be specific during the data collection period we were challenged by three independent problems those are (1) Unable to get laboratory TB registry log book in four health facilities, (2) the health facility representative of one health facility refused to participate in the study | 6 |
| 3a. Demographics and clinical characteristics of study participant on anti-TB treatment: First paragraph is a repetition of information provided in the “Study design and data collection” section. | Thank you for the suggestion. We made a correction per the suggestion. | 8 |
| 3b. Demographics and clinical characteristics of study participant on anti-TB treatment: Was the Xpert MTB/RIF assay used to diagnose pTB as well as EPTB? | Thank you for the curiosity. According to the data collected from the TB DOTs register we have seen that two EPTB cases were diagnosed by Xpert MTB/RIF assay, however, we didn’t get extra-pulmonary samples diagnosed by Xpert MTB/RIF assay from the TB laboratory register. | 8 |
| **Reviewer 2 COMMENT** | **AUTHOR RESPONSE** | **PAGE NUMBER** |
| The authors have reviewed retrospective data since the implementation of the Xpert assay for TB diagnosis at participating facilities in Addis Ababa. The aim was to determine the impact of Xpert on TB diagnosis compared to smear microscopy, which is expected as the sensitivity far exceeds that microscopy. Secondly, they further assessed the impact on treatment outcomes since the introduction of the technology - measuring the impact is vague as there are several other factors to be considered for treatment outcome and attributing outcomes to a diagnostic may not be the conclusive. The authors should have rather assessed impact on treatment initiation rather than outcome. The focus of the papers starts shifting between different variables not specifically linked to the Xpert testing. | Thank you for the comments. It is totally acceptable. In this study we have seen that an increased number of TB cases were diagnosed since the introduction of GeneXpert system for the diagnosis of all presumptive TB cases at the participating health facilities in Addis Ababa. There might be several factors that negatively or positively affect the treatment outcome; however, our intension was to see the trends in number of TB cases detected and the positive treatment outcome in the study period compared to the years before the introduction of GeneXpert system. Since as it is clearly said in the comment, there could be several other factors. So, we accept this comment and kept as one of the study limitation in the last paragraph of discussion section. | 15 |
| The paper structure needs review to improve the flow of information to provide the supporting evidence showing the impact of Xpert on both TB diagnosis and treatment outcomes. | Thank you for the suggestion. We made a correction per the suggestion. | All |
