# Supplementary material for: Impact of the use of GeneXpert on TB diagnosis and anti-TB treatment outcome at health facilities in Addis Ababa, Ethiopia in the post-millennium development years
Source: PLoS One. 2023 Aug 25;18(8):e0289917. doi: 10.1371/journal.pone.0289917 (PMC10456184; doi:10.1371/journal.pone.0289917)
Supplement: Attachment — Submitted filename: Response to Reviewers.doc [file pone.0289917.s003.doc]

**Letter to the academic editor and reviewers**

**Subject: Submission of a revised manuscript**

**Title: Impact of GeneXpert on TB diagnosis and anti-TB treatment outcome at health facilities in Addis Ababa, Ethiopia: in the post MDG years**

**Manuscript ID: PONE-D-21-40508R1**

Dear the academic editor and reviewer(s),

Thank you very much for your informative and constructive comments on the manuscript entitled “Impact of GeneXpert on TB Diagnosis and Anti-TB Treatment Outcome at Health Facilities in Addis Ababa, Ethiopia: In the Post MDG Years”. We also appreciate you for giving us the opportunity to revise our manuscript and correct errors in the previous version. We addressed all the comments one by one as seen in the table below.

| **Comment** | **Author Response** | **Page Number** |
| --- | --- | --- |
| 1. The introduction speaks of the ability of the GeneXpert to detect rifampicin resistance but these data are not included and it is unclear how the presence of RR is correlated with other measures. This is a serious flaw throughout the manuscript. | We, the authors, would like to acknowledge your concern. When we evaluate the treatment outcome of TB patients receiving anti-TB treatment at the selected health facilities, 65 patients have been transferred to MDR treatment sites because they are diagnosed as RR cases. This is the added value of Xpert MTB/RIF Assay that saves the transferred patients by early diagnosis and also prevented the unnecessary wastage of TB medicines. | Page 10 |
| 2. The methodology is incomplete - please discuss the specimens utilized (especially for the 0-14 age range) - were gastric washings, urine, blood and other samples included or just sputa. What was the failure rate of the equipment and what controls and monitoring were run. What was the reflex testing for culture and did any samples test positive on culture but negative on microscopy or GeneXpert. What was the clinical correlation in these cases. | Thank you for the suggestion. We made a correction per the suggestion. As indicated in page 3 of the manuscript, since 2017, the GeneXpert system is in use in the capital city of Ethiopia to diagnose all presumptive TB cases presented with signs and symptoms for TB. Which means, before 2017, the first diagnostic method in use was AFB Smear Microscopy. Therefore, we aimed only to see the impact of Xpert MTB/RIF assay on the number of TB cases detected in those years and which had a direct impact on the treatment outcome. To be specific in this study we did not intend to perform any tests rather we collected a retrospective data from the selected health facilities of Addis Ababa. The specimen type available on records for all age group is only sputa and also records of TB culture was not found. For better clarification as commented on methodology part under “Study design, data collection and ethical review” page 4 - we added a sentence which describes the sample source was sputa. Line 6 and 7. | Page 3 and 4 |
| 3. Treatment outcome is defined as successful if the patient was cured or the treatment was completed. Please clarify how "cure" was defined in the 27.3% of patients who were cured. In the high number of individuals who completed treatment only, what was the outcome if they were not cured? What is the definition of treatment failure (were they bacteriologically still positive) | Thank you for the comment.  Operational definitions have been kept for these terms in the methodology part page 5. | Page 5 |
| 4. The age range is suggestive of an economically active population. Was there any association with economic activity. The HIV statistics should be clarified in the context of the Ethiopian statistics as well. | Thank you for the comments. Correction has been made on the age range “15-34” as “15-44”. The HIV co-infection status is clarified against studies conducted in different cities/towns in Ethiopia like in Addis Ababa, Asela and Debre Tabor as indicated on page 14 in paragraph 3. | Page 6 and 13 |
| 5. Please change HIV reactive and unreactive to infected. Please also indicate where known whether patients were virologically suppressed or on ART (if known) and indicate whether this has a relationship to treatment outcome. | Thank you for the comments and we made corrections per the comments on table 3, 4 and 5 as well as in the respective result summary paragraphs. In this study, we have collected retrospective data from TB lab and treatment logbook in which the virological suppression status of HIV-infected patients was not recorded except HIV reactive and non-reactive. | Page 8-11 |
| 6. Please confirm what transfer in indicates | Thank you for the suggestion.  Transfer-in is a TB patient category who has been transferred from another health facility at any time to initiate or continue the treatment for the convenient of TB patient. Based on the comment, its definition for clarity has been kept under operational definitions in the methodology part, page 5. | Page 5 |
| 7. Were all treated patients and retreated patients on a standard 6month regimen? If not, how many were on second line therapy. Were these patients admitted? | Thank you for the comments.   - All treated and retreated patients included in the treatment outcome analysis were on a standard 6-month regimen. - 65 patients who are not included in the treatment outcome analysis as indicated under table 4 on page 10 were transferred out to other health facilities for second-line therapy which are not included in this study since their treatment records are not found in the study health facilities. - In general, at national level, TB patients for first line TB treatment are not admitted whereas those who take second line TB treatment are admitted. | Page 10 |
| 8. The following should be included in the discussion: why was there a slight male preponderance, can you hypothesise as to the age spread, can you comment on the efficacy of the test, can you comment on the acuracy of the test, can you link treatment response to HIV status, is there any other comorbid condition (diabetes, hypertension, obesity, cancer etc) which may explain the results. If these data are not available, that represents a significant limitations which should be addressed. In short there is a lack of explanation of the relevant findings which must be addressed. | Thank you for the comments. We made corrections per the comments as indicated on page 13 last paragraph. The secondary data that we collected do not enable us to calculate the efficacy and accuracy of the test and it was not our objective initially. In addition, we didn’t get comorbidities other than HIV co-infection in the TB lab and treatment logbooks. So, we kept the absence of other comorbidities data as a limitation as commented on page 15 last paragraphs. | Page 13 and 15 |
| 9. A minor comment is that the manuscript still requires significant English language editing. | Thank you for the comments and we made corrections per the comments. | All |
