# Supplementary material for: Impact of the use of GeneXpert on TB diagnosis and anti-TB treatment outcome at health facilities in Addis Ababa, Ethiopia in the post-millennium development years
Source: PLoS One. 2023 Aug 25;18(8):e0289917. doi: 10.1371/journal.pone.0289917 (PMC10456184; doi:10.1371/journal.pone.0289917)
Supplement: Attachment — Submitted filename: Response to Reviewers.doc [file pone.0289917.s004.doc]

**Letter to the academic editor and reviewers**

**Subject: Submission of a revised manuscript**

**Title: Impact of GeneXpert on TB diagnosis and anti-TB treatment outcome at health facilities in Addis Ababa, Ethiopia: in the post-MDG years**

**Manuscript ID: PONE-D-21-40508R2**

Dear academic editor and reviewer(s),

Thank you very much for your informative and constructive comments on the manuscript entitled “Impact of GeneXpert on TB Diagnosis and Anti-TB Treatment Outcome at Health Facilities in Addis Ababa, Ethiopia: In the Post MDG Years”. We also appreciate for giving us the opportunity to submit the revised version of our manuscript. We addressed all the comments one by one as seen in the table below.

| **Comment** | **Author Response** | **Page Number** |
| --- | --- | --- |
| 1. Can the author explain the discrepancy between the date of the implementation of GeneXpert (2017) and the data which include 2015 and 2016 | Thank you for the comments.  We, the authors, would like to make clear the discrepancy related to the implementation of GeneXpert in Ethiopia;   - It is true that the GeneXpert instruments were introduced in Ethiopia and in use since 2012 (Alemu et al., 2019), to diagnose only selected presumptive TB cases (Presumptive DR-TB, HIV +ve, Children; and EPTB), while the remaining cases with low-risk to DR-TB, HIV-VE, and adult were diagnosed by AFB microscopy based on the algorithm (FMoH Ethiopia, 2014). But, since 2017, GeneXpert system is in use to diagnose all presumptive TB cases presented with signs and symptoms of TB at any given health facility in Addis Ababa. - It is because of selective TB diagnosis made by GeneXpert instruments were available in the years 2015 and 2016. |  |
| 2. Can the author confirm the methodology - here a flow diagram may be useful - outlining the sensitivity and specificity of microscopy as well as the exact staining and review process used | Thank you very much for the comments.  The sensitivity and specificity were not assessed since, in this investigation, we collected retrospective data from health facilities in Addis Ababa.  Both staining techniques (Ziel-Neelsen staining (ZN) or fluorescent auramine staining (LED FM)) were in use in Addis Ababa but the health facilities do not keep track of which staining techniques were employed; instead, the record indicates that the diagnosis was performed using sputum smear microscopy. Due to the lack of clarity in the registration books, we are unable to determine the exact staining techniques. |  |
| 3. This manuscript still requires extensive English language editing | Thank you for the comments and suggestions, and we made corrections per the comments. |  |
| 4. Reviewer #3:  - Data in Table 1 shows an increase in TB testing (Total), I would like to know the reasons for the increase uptake from 4 635 in year 2015 to 17 572 in Year 2018. - Secondly, the method for diagnosing using smear, was it a single smear per participant? if so why do we see an increase in uptake as GeneXpert testing is introduced. | Thank you for the comments.   - It is true that since the GeneXpert instrument has been used for diagnosis of presumptive TB cases as a first-line test in the chosen health facilities in Addis Ababa, the number of presumptive TB cases diagnosed for TB has increased. The instrument's improved sensitivity and specificity, which increase the trust of clinicians working in the OPD, ease of use, the lack of a requirement for two sputum samples, and the suitability of the reagent/cartridge may all be contributing factors to the increased TB test. - Based on the recommendation of the guideline for clinical and programmatic management of TB and TB/HIV in Ethiopia, two sputum samples (Spot-Spot) are required for AFB Microscopy, and one sputum smear positive result confirms TB diagnosis. In this study, we collected the final AFB microscopy result of the patients from the laboratory registration book. |  |
| 5. Reviewer #4: - There are serious issues with style and grammar that affect comprehension and require editing   - There is inconsistency between information given in background and results: the background states that GXP was introduced in 2017, yet GXP data is presented from 2015 onwards  - The background lacks information on microscopy and GXP in terms of performance data/limit of detection  - The method section is flawed; there is no information on MTB testing algorithms in the laboratories; no information on which AFB microscopy method was used; no information on treatment regimens for MDR TB; extracted data and analysis thereof is not clearly stated  - The results section doesn't mention proportion of patients on DS or MDR treatment; patients with extra-pulmonary TB are included yet the methods section only mentions sputum samples-- how were these diagnosed and from which samples?; the impact of the GXP introduction on treatment outcomes is not mentioned in results and should be elaborated on; relevance of adding data for various 'sub-cities' not clear unless of local interest | Thank you for the comments.     - Thank you for the comments and we made corrections per the comments. - It is true that the GeneXpert instruments were introduced in Ethiopia and in use since 2012 (Alemu et al., 2019), to diagnose only selected presumptive TB cases (Presumptive DR-TB, HIV +ve, Children; and EPTB), while the remaining cases with low-risk to DR-TB, HIV-VE, and Adult are diagnosed by AFB microscopy based on the algorithm (FMoH Ethiopia, 2014). But, since 2017, GeneXpert system is in use to diagnose all presumptive TB cases presented with signs and symptoms of TB at any given health facility in Addis Ababa. It is because of selective TB diagnoses made by GeneXpert instruments were available in the years 2015 and 2016. - The performance data is included on page 3 in the last paragraph (we added a sentence which describes the performance of AFB smear microscopy) - Both staining techniques (Ziel-Neelsen staining (ZN) or fluorescent auramine staining (LED FM)) are in use in Addis Ababa, but the health facilities do not keep track of which staining techniques were employed; instead, the record indicates that the diagnosis was performed using sputum smear microscopy. Due to the lack of clarity in the laboratory registration books, we are unable to determine the exact staining techniques. - 65 MDR patients who were moved to the MDR Treatment Initiating Center (TIC) for second-line therapy and are not included in this study, as noted under Table 4 on page 10. Therefore, we were unable to obtain their treatment records. As a result, MDR patients are excluded from the treatment outcomes analysis. - Clinical diagnosis was the main method of diagnosis for extra-pulmonary TB patients. Based on the recorded data only 10 (0.3%) of the 3694 extra-pulmonary TB patients had laboratory diagnostic information, The data record simply states that the diagnosis was made using either smear microscopy or GeneXpert, the health facilities did not keep track of the sample types utilized. That is why we don’t give due attention on the diagnosis of EPTB and we are unable to pinpoint the precise sample type utilized. - The result section on page 10 and lines 10^th^ and 11^th^ discuss the impact of the GXP introduction on treatment results. - We appreciate your concern and your suggestion, adding data for various 'sub-cities' will be pertinent to local interest. |  |
