# Supplementary material for: Impact of the use of GeneXpert on TB diagnosis and anti-TB treatment outcome at health facilities in Addis Ababa, Ethiopia in the post-millennium development years
Source: PLoS One. 2023 Aug 25;18(8):e0289917. doi: 10.1371/journal.pone.0289917 (PMC10456184; doi:10.1371/journal.pone.0289917)
Supplement: Attachment — Submitted filename: Response to Reviewers.doc [file pone.0289917.s006.doc]

**Letter to the academic editor and reviewers**

**Subject: Submission of a revised manuscript**

**Title: Impact of GeneXpert on TB diagnosis and anti-TB treatment outcome at health facilities in Addis Ababa, Ethiopia: in the post-MDG years**

**Manuscript ID: PONE-D-21-40508R3**

Dear academic editor and reviewer(s),

Thank you very much for your informative and constructive comments on the manuscript entitled “Impact of GeneXpert on TB Diagnosis and Anti-TB Treatment Outcome at Health Facilities in Addis Ababa, Ethiopia: In the Post MDG Years”. We also appreciate for giving us the opportunity to submit the revised version of our manuscript. We addressed all the comments one by one as seen in the table below.

| **Comment** | **Author Response** | **Page Number** |
| --- | --- | --- |
| Journal Requirements: - Please review your reference list to ensure that it is complete and correct. | Thank you for the comments and suggestions, and we made corrections per the comments and suggestions.  The year of publication for reference number 13 has been changed from 2019 to 2014 because the year of publication was incorrect. | Page 3, 5 and 16 |
| Reviewer #4: - I would suggest additional work on the following issues   - Style and grammar require further review, there are several phrases and words that are not idiomatic or incorrect. Please also review the tense in which the various sections are written. Some examples highlighted in yellow in attachment. - Not all abbreviations are explained in the text (see PTBN and PTBP under ‘demographics and clinical characteristics’). - Should the sentence in the discussion in the third paragraph ‘Male patients who were diagnosed with TB had higher odds of being positive..’ say ‘screened’ rather than ‘diagnosed’? A positive test would lead to the diagnosis, the sentence as it stands doesn’t seem to make sense. This appears again on another occasion in the discussion. - The discussion still lacks a clear structure and a logical flow, and should focus more on the main aims of the study which were to show a link between increase in Xpert testing and TB diagnosis and treatment outcomes. The link between introduction of Xpert and improved treatment outcomes is tenous and does not appear to be supported by the data: the treatment outcome group that was analysed appears to include patients that were diagnosed clinically and a large number of patients with extra-pulmonary TB who were also not diagnosed with Xpert it seems (or ?unknown how they are diagnosed). Yet the discussion states that Xpert ‘brought an improvement in…success rate of treatment outcomes’. Please could you clarify how you came to this conclusion? There are recent review articles that have not shown a strong link, ie F Haraka 2021 ‘Impact of the diagnostic test Xpert MTB/RIF on patient outcomes for tuberculosis’ and G Di Tanna 2019 ‘Effect of Xpert MTB/RIF on clinical outcomes in routine care setting: individual patient data meta-analysis’. - You do mention in your limitations that other factors that may have had an impact on treatment outcomes were not taken into consideration; I feel this has to be expanded on as it is very relevant. It is mentioned that patients with drug resistant TB were excluded from the analysis so any advantage of picking up rifampicin resistance using the Xpert cartridge on treatment outcomes will not be assessed if I am correct? | - Thank you for the comments and suggestions, and we made corrections per the comments and suggestions forwarded - Thank you for the suggestions, and we made corrections per suggestions forwarded - Thank you for the comments and suggestions, and we made amendment as per the suggestion - Thank you for the comments and suggestions, and we made corrections per the suggestion. Regarding the conclusions made on the impact of Xpert MTB/RIF Assay on the treatment success rate;   - It is true that this study has limitation related assessing some factors or actions taken in health facilities which might have impact for the improved treatment outcome; however, we have seen that the number of presumptive TB cases screened and diagnosed since the introduction of the Xpert MTB/RIF Assay were significantly increased and also an increased treatment success rate and 30% drop in death rate proves that the introduction of Xpert MTB/RIF Assay as a first diagnostic method for presumptive   - Additionally, among bacteriologically confirmed pulmonary TB cases, the percent of cured patients were increased to 34.4% in 2018 compared to 22.8% in 2015, since the cure treatment outcome evaluation requires confirmation by diagnostic tests Xpert MTB/RIF assay. - Thank you for the suggestions, and we made corrections per the suggestion that is exclusion of patients with drug resistant TB is included in the limitation section | Page 16 |
| Reviewer #5: I think that this is an important subject and provides valuable information on implementation of Gene Xpert, however, there are still a number of errors that need to be addressed for example   - In the second paragraph of the discussion, they state that there has been a 4 fold increase in identified TB cases, I think the correct term is, confirmed TB cases have increased 4 fold. - Similarly, in paragraph 5 of the discussion they state that the cure rate pulmonary TB was 92.9%, they previously should either clarify that they are referring to the PTBP or cite the actual pulmonary TB cure rate which was 27.3%. While the authors have addressed the comments raised by the reviewers there are still some errors that need to be addressed. | Thank you for the comments and suggestions   - Thank you for the comments and suggestions. We made corrections per the comments. - Thank you for the comments and suggestions. The variation in result among the cured PTBP patient observed is due to the denominator used that means;   - We used the entire number of patients enrolled as a denominator for the result narrative section on page 9 under ‘demographics and clinical characteristics,’ and for the result table section on table 3, we used the total number of PTBP patients as a denominator. | Page 15 |
